# Supplementary material for: Protocol for Project Fizzyo, an analytic longitudinal observational cohort study of physiotherapy for children and young people with cystic fibrosis, with interrupted time-series design
Source: BMJ Open. 2020 Oct 7;10(10):e039587. doi: 10.1136/bmjopen-2020-039587 (PMC7542954; doi:10.1136/bmjopen-2020-039587)
Supplement: Author's manuscript [file bmjopen-2020-039587.draft_revisions.pdf]

BMJ Open is committed to open peer review. As part of this commitment we make the peer review history of every article we publish publicly available.

When an article is published we post the peer reviewers' comments and the authors' responses online. We also post the versions of the paper that were used during peer review. These are the versions that the peer review comments apply to.

The versions of the paper that follow are the versions that were submitted during the peer review process. They are not the versions of record or the final published versions. They should not be cited or distributed as the published version of this manuscript.

BMJ Open is an open access journal and the full, final, typeset and author-corrected version of record of the manuscript is available on our site with no access controls, subscription charges or pay-per-view fees (http://bmjopen.bmj.com).

If you have any questions on BMJ Open's open peer review process please email info.bmjopen@bmj.com

BMJ Open

Protocol for Project Fizzyo, an analytic longitudinal observational cohort study of physiotherapy for children and young people with cystic fibrosis, with interrupted time series design

Journal:	*BMJ Open*
Manuscript ID	bmjopen-2020-039587
Article Type:	Protocol
Date Submitted by the Author:	20-Apr-2020

Complete List of Authors:	Raywood, Emma; University College London Institute of Child Health, Physiotherapy, Respiratory, Critical Care and Anaesthesia Section, Infection, Inflammation and Immunity Programme,<br>Douglas, Helen; University College London Institute of Child Health, Physiotherapy, Respiratory, Critical Care and Anaesthesia Section, Infection, Inflammation and Immunity Programme,<br>Kapoor, Kunal; University College London Institute of Child Health, Physiotherapy, Respiratory, Critical Care and Anaesthesia Section, Infection, Inflammation and Immunity Programme,<br>Filipow, Nicole; University College London Institute of Child Health, Physiotherapy, Respiratory, Critical Care and Anaesthesia Section, Infection, Inflammation and Immunity Programme,<br>Murray, Nicky; Royal Brompton and Harefield NHS Foundation Trust, Paediatric Cystic Fibrosis Unit,<br>O'Connor, Rachel; Barts Health NHS Trust, Paediatric Cystic Fibrosis Centre, Royal London Hospital,<br>Stott, Lee; Microsoft UK Ltd - Reading, Commercial Software Engineering,<br>Saul, Greg; Microsoft Research Ltd, Microsoft Research Lab,<br>Kuzhagaliyev, Tim; University College London Department of Computer Science, Computer Science<br>Davies, Gwyneth ; University College London Institute of Child Health, Respiratory Critical Care and Anaesthesia section, Infection Inflammation and Immunity Programme,<br>Liakhovich, Olga; Microsoft UK Ltd - Reading, Commercial Software Engineering,<br>Van Schaik, Tempest; Microsoft UK Ltd - Reading, Commercial Software Engineering,<br>Furtuna, Bianca; Microsoft UK Ltd - Reading, Commercial Software Engineering,<br>Booth, John; Great Ormond Street Hospital For Children NHS Foundation Trust, Digital Research Environment,<br>Shannon, Harriet; University College London Institute of Child Health, Physiotherapy, Respiratory, Critical Care and Anaesthesia Section, Infection, Inflammation and Immunity Programme,<br>Bryon, Mandy; Great Ormond Street Hospital For Children NHS Foundation Trust, Department of Paediatric Psychology,<br>Main, Eleanor; University College London Institute of Child Health,

	Physiotherapy, Respiratory, Critical Care and Anaesthesia Section, Infection, Inflammation and Immunity Programme,
Keywords:	Cystic fibrosis < THORACIC MEDICINE, Paediatric thoracic medicine < PAEDIATRICS, BIOTECHNOLOGY & BIOINFORMATICS, Protocols & guidelines < HEALTH SERVICES ADMINISTRATION & MANAGEMENT

SCHOLARONE™
Manuscripts

I, the Submitting Author has the right to grant and does grant on behalf of all authors of the Work (as defined in the below author licence), an exclusive licence and/or a non-exclusive licence for contributions from authors who are: i) UK Crown employees; ii) where BMJ has agreed a CC-BY licence shall apply, and/or iii) in accordance with the terms applicable for US Federal Government officers or employees acting as part of their official duties; on a worldwide, perpetual, irrevocable, royalty-free basis to BMJ Publishing Group Ltd ("BMJ") its licensees and where the relevant Journal is co-owned by BMJ to the co-owners of the Journal, to publish the Work in this journal and any other BMJ products and to exploit all rights, as set out in our licence.

The Submitting Author accepts and understands that any supply made under these terms is made by BMJ to the Submitting Author unless you are acting as an employee on behalf of your employer or a postgraduate student of an affiliated institution which is paying any applicable article publishing charge ("APC") for Open Access articles. Where the Submitting Author wishes to make the Work available on an Open Access basis (and intends to pay the relevant APC), the terms of reuse of such Open Access shall be governed by a Creative Commons licence – details of these licences and which Creative Commons licence will apply to this Work are set out in our licence referred to above.

Other than as permitted in any relevant BMJ Author's Self Archiving Policies, I confirm this Work has not been accepted for publication elsewhere, is not being considered for publication elsewhere and does not duplicate material already published. I confirm all authors consent to publication of this Work and authorise the granting of this licence.

For peer review only

Protocol for Project Fizzyo, an analytic longitudinal observational cohort study of physiotherapy for children and young people with cystic fibrosis, with interrupted time series design

Authors: Emma Raywood[1] e.raywood@ucl.ac.uk, Helen Douglas[1] h.douglas@ucl.ac.uk, Kunal Kapoor[1] k.kapoor@ucl.ac.uk, Nicole Filipow[1] nicole.filipow.18@ucl.ac.uk, Nicky Murray[2] nickymurray@nhs.net, Rachel O'Connor[3] rachel.oconnor1@nhs.net, Lee Stott[4] leestott@microsoft.com, Greg Saul[5] gregsaul@gmail.com, Tim Kuzhagaliyev[6] tim.kuzh@gmail.com, Gwyneth Davies[1] gwyneth.davies@ucl.ac.uk, Olga Liakhovich[4] olga.liakhovich@microsoft.com, Tempest Van Schaik[4] tempest.van@microsoft.com, Bianca Furtuna[4] bianca.furtuna@microsoft.com, John Booth[7] john.booth@gosh.nhs.uk, Harriet Shannon[1] h.shannon@ucl.ac.uk, Mandy Bryon[8] mandy.bryon@gosh.nhs.uk, and Eleanor Main[1] e.main@ucl.ac.uk.

Affiliations: [1] Physiotherapy, Respiratory Critical Care and Anaesthesia section, Infection Inflammation and Immunity Programme, University College London Great Ormond Street Institute of Child Health, London, UK, [2] Paediatric Cystic Fibrosis Unit, Royal Brompton Hospital, Royal Brompton & Harefield NHS Foundation Trust, London, UK, [3] Paediatric Cystic Fibrosis Centre, Royal London Hospital, Barts Health NHS Trust, London, UK, [4] Commercial Software Engineering, Microsoft UK, London/Reading UK, [5] Microsoft Research Lab, Microsoft Research, Cambridge, UK, [6] Department of Computer Science, University College London, London, UK. [7] Digital Research Environment, Great Ormond Street Hospital for Children NHS Foundation Trust, London, UK, [8] Department of Paediatric Psychology, Great Ormond Street Hospital for Children NHS Foundation Trust, London, UK,

Corresponding Author: Emma Raywood, e.raywood@ucl.ac.uk Physiotherapy, Respiratory Critical Care and Anaesthesia section, Infection Inflammation and Immunity Programme, University College London Great Ormond Street Institute of Child Health, London, UK.

Keywords: Cystic fibrosis, Data science, Paediatrics, Physical activity, Physiotherapy

Word Count: 3689 words

ABSTRACT

Introduction: Daily physiotherapy is believed to mitigate the progression of cystic fibrosis (CF) lung disease. However, physiotherapy airway clearance techniques (ACTs) are burdensome and the evidence guiding practice remains weak. This paper describes the protocol for Project Fizzyo, using innovative technology and analysis methods to remotely capture longitudinal daily data from physiotherapy treatments to measure adherence and prospectively evaluate associations with clinical outcomes.

Methods and analysis: A cohort of 145 children and young people with CF aged 6-16 years were recruited. Each participant records their usual physiotherapy sessions daily for 16 months, using remote monitoring sensors: 1) a bespoke ACT sensor, inserted into their usual ACT device and 2) a Fitbit Alta HR™ activity tracker. Real time breath pressure during ACTs, and heart rate and daily step counts (Fitbit) are regularly synced using specific software applications. An interrupted time series design facilitates evaluation of ACT interventions (feedback and ACT-driven gaming).

Baseline, mid and end-point assessments of spirometry, exercise capacity, and quality of life as well as longitudinal clinical record data are also collected.

This large dataset will be analysed in R using big data analytics approaches. Distinct ACT and physical activity adherence profiles will be identified using cluster analysis to define groups of individuals based on measured characteristics and any relationships to clinical profiles will be assessed. Changes in adherence to physiotherapy over time or in relation to ACT gaming or feedback will be quantified and evaluated in relation to clinical outcomes.

Ethics and dissemination: Ethical approval was granted by the London-Brighton and Sussex, NREC (18/LO/1038 protocol v3.1). The ISRCTN registry reference is 51624752. Findings will be disseminated via peer-reviewed publications, at conferences, and via CF clinical networks. The statistical code, will be published in the Fizzyo GitHub repository and the dataset stored and available through the GOSH DRE.

ARTICLE SUMMARY

Strengths and limitations of this study

- This research is directly related to four of the James Lind Alliance top ten research priorities for CF; the application of new technology, evidence for effectiveness of current therapies, reduction of treatment burden, and the relationship of exercise and ACT.
- Participants do not have to change their daily physiotherapy techniques from usual routine care; the observational design captures objective real-world data.
- The novel use of remote monitoring for physiotherapy has the potential to provide evidence to guide ACT practice where randomised controlled trials have failed.
- The development of data infrastructure including a pipeline for recording, syncing, processing and analysis of remote monitoring and clinical data is vital for the future use of these technologies.

For peer review only

INTRODUCTION

Cystic Fibrosis (CF) is a life-limiting inherited condition, which affects over 10,000 people in the UK. Despite recent treatment advances, CF remains progressive and incurable. People with CF are susceptible to repeated respiratory infections due to thick respiratory mucus, which can lead to irreversible lung damage. Daily treatment to slow the progression of lung disease, and therefore the onset of respiratory failure, is the primary aim of almost all current CF therapies,[1]. Daily physiotherapy, including airway clearance techniques (ACTs), nebulised therapies, physical activity and exercise, is believed to mitigate the progression of CF lung disease,[2].

People with CF undertake a median of ten different concurrent treatments that take an average of two hours each day,[3]. This high daily treatment burden (and high cost of care) from childhood has become a driver for research to maximise effective care, and minimise unnecessary therapies. Reducing treatment burden has been identified as the top CF research priority by the James Lind Alliance,[4]. Follow-up research into treatment burden found that although ACTs were perceived as very important, they were considered to be the most burdensome daily treatment. ACTs are stressful for people with CF and their families, and adherence can be low; 70% of the people with CF questioned regularly missed some of their daily treatments, most commonly skipping ACTs or nebulised therapies,[3]. Another study found those with the lowest adherence to chest physiotherapy had worse lung function, more exacerbations and consequently had higher health costs,[5].

Another of the James Lind Alliance top ten research priorities for CF is to identify which specific therapies can delay and prevent progression of lung disease in early life. Despite over 70 years of ACTs in routine CF clinical practice, the evidence base to guide treatment remains weak. A number of challenges exist for researchers in the field; a plethora of physiotherapy ACTs and devices exist, providing a bewildering choice for therapists and patients, with the long term effects of different devices, techniques or non-adherence being poorly understood. Traditional research methods (including randomised controlled trials) have failed to produce credible evidence for optimal ACT type, dose, frequency or duration,[6, 7]. Established solitary outcome measures (e.g. FEV_1) are insensitive to change in mild CF lung disease, and are not useful end points for physiotherapy clinical trials. Furthermore blinding is not possible, patient or practitioner preferences can confound results of trials and ethical concerns about the complete removal of ACTs persist,[2]. There is evidence that patients with the lowest self-reported physical activity levels have poorer health,[8] but the use of exercise as an ACT remains controversial,[9] despite the fact it is a popular idea with patients,[4].

Advances in technology, including increased use of electronically chipped devices, electronic patient records and the growth of big data analytics for large and diverse data sets, are providing fresh avenues and opportunities for physiotherapy research. These may facilitate clarity and certainty about effective therapies and help to reduce treatment burden. Big data techniques, including machine learning and unsupervised clustering, have been shown to be useful for analysis of healthcare data, which is often complex, un-structured and from different sources. However, they are yet to be used to build credible evidence to guide physiotherapy in people with CF.

Project Fizzyo

This paper describes the protocol for Project Fizzyo (ISRCTN: 51624752), which uses innovative technology to capture detailed longitudinal data from children and young people with CF (CYPwCF) undertaking daily physiotherapy treatments. Real time breath pressure during ACTs are captured daily, as well as heart rate and footstep data during physical activity. The interrupted time series study design facilitates evaluation of interventions such as ACT feedback and ACT-driven gaming.

For peer review only - http://bmjopen.bmj.com/site/about/guidelines.xhtml

The aims of Project Fizzyo are 1) to monitor and understand adherence to daily ACT prescriptions, 2) evaluate associations between ACT adherence and clinical outcomes under different study conditions (including feedback and gaming), 3) to monitor and understand daily patterns of physical activity in relation to published recommendations, and evaluate any associations with clinical outcomes, and 4) to use big data analytics to seek out important data signals in relation to optimising ACT and exercise prescriptions for CYPwCF.

METHODS

Study design

Project Fizzyo is an analytic longitudinal observational cohort study with an interrupted time series design. Clinically prescribed ACT or exercise prescriptions for individual participants are continued as normal for 16 months during the study. Each participant uses two remote monitoring sensors to record data daily. These are: 1) a bespoke Fizzyo ACT breath pressure sensor, inserted into their regular ACT device (an Acapella® Choice, Aerobika®, AstraTech® PEP or Pari PEP™), and 2) a Fitbit Alta HR™ activity tracker (Fitbit Inc, San Francisco, USA) to record daily physical activity (heart rate and steps). Participants synchronise (sync) data using apps for each sensor, on a tablet computer provided for the study.

ACT feedback (daily number of breaths) and ACT-driven computer gaming are introduced and removed in an interrupted time series design (figure 1), via the specially developed Fizzyo app. Baseline ACT patterns are recorded during months 0-2 (Standard phase), then sequential introduction of ACT feedback (during months 2-14: Feedback phase) and ACT-driven gaming (during months 4-12: Gaming phase) will allow any effect on breathing patterns, adherence or clinical outcomes to be observed. The removal of gaming (at month 12) and feedback (at month 14) will facilitate observation of lasting, temporary or feedback/game dependent behaviour changes.

Patients and their families partnered with us for the choice and development of the sensors, apps and games from a patient's perspective as well as the study design and information materials.

Participants

To be eligible participants at recruitment were: 1) aged 6-16 years, 2) diagnosed with CF and under the care of a participating London paediatric CF centre (either Great Ormond Street Hospital (GOSH), the Royal London Hospital (RLH) or the Royal Brompton Hospital (RBH), including shared care patients), and 3) using one of four ACT devices which are compatible with the ACT sensor (Acapella, Aerobika, Astratech PEP or Pari PEP), at least once a day as part of routine ACTs.

Patients were excluded if 1) they or their parents did not or could not provide informed consent, 2) they were not using or prescribed one of the 4 specific ACT devices as part of their routine daily treatment, 3) had undergone lung transplantation or 4) had a clinically significant medical condition other than CF.

Children meeting the eligibility criteria were approached by their clinical team with the study information sheets. Participant information sheets affirm that no direct benefits are promised to participants, although they may find the computer games help them to enjoy and engage with airway clearance. After 16 months the study will end for each participant and the ACT sensor and Fitbit will be returned. Participants can choose to keep the devices, with restored games and feedback for a short period at the end of their participation, on the condition they continue to sync data until the remaining period of data collection is concluded for all participants (December 2020).

For peer review only - http://bmjopen.bmj.com/site/about/guidelines.xhtml

Participants were recruited from September 2018 to July 2019. The recruitment window was scheduled to be open for 6 months per site, though for the first and largest recruiting site (GOSH) an extra month was added to allow for a period of pilot data collection to resolve any early technical issues. The initial recruitment target of 160 participants was reduced to 145 due to manufacturing delays for the bespoke Fizzyo sensor, which was not a threat to meeting the aims of the study. This is an observational study, with 145 CYPwCF representing the largest CF population sample in any physiotherapy study undertaken in Europe to date. Daily data from these children over 16 months will provide sufficient precision of estimation in evaluating the signals of adherence to therapy.

The number of children (recruitment periods) at each hospital were: at GOSH 75, (10/09/2018-16/04/2019), RBH 40, (30/11/2018-31/05/2019) and RLH 30 (03/01-01/07/2019). Data collection is ongoing and is predicted to end in December 2020.

Data collection

Following informed consent from a parent/guardian or, when appropriate, the participant if >15years, with a member of the research team, each participant received identical study equipment (table 1, figure 2) to record and synchronise data. Each participant was issued a unique anonymous Microsoft log-in account with a strong password (managed by the study team) for logging into the tablet, Fitbit app (Fitbit Inc, San Francisco USA) and bespoke Fizzyo app. Instructions for daily use during the 16-month data collection period and technical support contact details were also provided.

Table 1 Study equipment for participants

	ACT sensor	Activity tracker	Tablet computer
**Description**	Bespoke Fizzyo Sensor.<br>Wireless battery powered pressure sensor that attaches to ACT devices to measure pressure during ACT.	Fitbit Alta HR™.<br>Wrist-worn activity tracker with heart rate sensor to record daily physical activity.	Linx™ 12x64 Windows 10 tablet computer.<br>Required to host remote monitoring apps and games.
**Features**	• Microelectromechanical system (MEMS) based piezoresistive sensor.<br>• Two Buttons for power on/off and game control input.<br>• 1MB flash based storage for approximately 227 hours of data.<br>• 7 days use from full charge. Full charge: 70 minutes.	• OLED customisable display with clock face.<br>• Photoplethysmographic PurePulse® technology heart rate sensor.<br>• 3-axis accelerometer movement detection.<br>• Memory capacity for 7 days of full data.<br>• 7 days use from full charge. Full charge: 1-2 hours.	• Intel Atom x5 processor.<br>• 4GB RAM.<br>• Bluetooth 4.0.<br>• Wi-Fi 802.11.<br>• 64GB memory storage.<br>• 5-7 hours use from full charge. Full charge: 3 hours.
**Data collected**	• Time-stamped ACT pressure data (10Hz).	• Heart rate (variable sampling frequency; 6-30times per minute),<br>• Steps (per minute).	• Extraction and transmission of Fizzyo sensor and Fitbit data via sync with apps.<br>• Gaming data.
**App Details**	**Fizzyo app (FizzyoHub)**<br>• Developed using Visual Studio for Windows 10,<br>• Bluetooth syncing of the Fizzyo ACT sensor .	**Fitbit app (for Windows 10)**<br>• Developed by Fitbit Inc.<br>• Bluetooth syncing of the Alta HR.	**Windows store app**<br>• Required to install and update Fitbit app and Fizzyo ACT games.

For peer review only

	• *Standard phase*, syncing only,<br>• *Feedback phase*, daily and monthly breath count graphs enabled,<br>• *Gaming phase,* games (developed in Unity, hosted in Microsoft store) able to be installed and played.	• Patient facing dashboard displaying daily and historical graphs of step and activity patterns.<br>• Feedback on progress against daily and longer term goals.	

At recruitment, baseline assessments of lung function (spirometry: FEV_1, FVC, $FEF_{25\text{-}75}$), exercise capacity (10m modified shuttle walk test, with a Polar H10™ heart rate monitor), a quality of life questionnaire (Revised CF Questionnaire (CFQ-R)) and a physiotherapy questionnaire were completed by participants with a member of the study team digitally via REDCap. These measurements are repeated midway (7-9 months, estimated completion by May 2020) and at the end (16-months, estimated completed in December 2020) of the study. Assessments are carried out on days when participants have a clinic appointment or routine hospital admission (not during exacerbation).

All data are pseudonymised, stored and analysed in the secure GOSH Digital Research Environment (DRE, Aridhia Inc, Edinburgh, UK). The DRE is a cloud-enabled, dedicated GOSH secondary use data store of medical data from electronic patient records, remote monitoring sensors or external databases. Embedded analytic tools enable approved researchers to perform statistical analysis within the DRE. Data encryption and transfer protocols and data sharing and processing agreements ensure data are processed in a responsible, lawful, secure and confidential way compliant with NHS information governance standards, the UK data protection act (2018) and the EU GDPR (2018).

Remote monitoring

Activity tracker

A Fitbit activity tracker was chosen as Fitbit Inc. allow developers to use an application programming interface (API) for extraction of processed granular heart rate and footstep data. The specific activity tracker model used for this study, the Fitbit Alta HR (released in 2017), was chosen with input from CYPwCF and their families at GOSH, from a range of Fitbit devices. The slim display is an appropriate size for the wrists of children from 6 years old and it is simple to use. It has a photoplethysmographic heart rate sensor for heart rate measurement, which is essential to physiologically quantify adherence to recommended minutes in daily moderate to vigorous physical activity (MVPA).

Participants are asked to wear the Fitbit during all waking hours except when bathing/swimming (not waterproof) and to sync data daily via Bluetooth using the Fitbit app, or at a minimum once per week: the memory capacity of the tracker. Participants are asked to charge the battery at least once per week or when notified by the watch/app. The standard Fitbit app provides feedback data, which is available for participants to view throughout the study. After participants sync their device, the study team extract anonymised data from the Fitbit cloud using the API. It is downloaded to the Fizzyo data cloud and then transferred securely into to the DRE (figure 2).

ACT sensor

A bespoke wireless sensor was developed for Project Fizzyo with involvement from CYPwCF and their families at GOSH, physiotherapy specialists and product design specialists. The sensor is battery

For peer review only

powered and clips on to compatible ACT devices (Acapella (figure 3), Aerobika, Astratech PEP or Pari PEP) in the same way that pressure manometers are clipped in during ACT training or use. It measures air pressure changes inside the physiotherapy ACT device during breathing.

Participants are instructed to use the sensor to record all ACT treatments during the study. To reduce the chance of data loss, participants are asked to sync data once per day using the Fizzyo app and charge the sensor at least once per week. Pressure data are stored on the sensor's encrypted memory chip (capacity 1Mb) and can be synchronised either immediately after treatment or stored to sync at a later time. Immediately upon syncing data to the Fizzyo cloud (figure 2) basic breath counting processing occurs (in the Fizzyo cloud), so breath count feedback can be displayed to participants within a few seconds of sync (during the feedback stage of the study only). Detailed breath count analysis is performed on cleaned pseudonymised data in the DRE.

ACT-driven gaming

Windows compatible games have been developed using a bespoke structured gaming framework (https://github.com/Fizzyo/FizzyoFramework-Unity) in Unity (Unity Technologies, San Francisco, USA), through collaboration with Microsoft, UCL and Abertay University students and children with CF at GOSH. The framework takes real time inputs during ACT treatments (expiratory pressure during breathing and a button press) from the Fizzyo sensor, and uses these to simulate standard joystick inputs.

Five bespoke games with different mechanics are available to participants (including Qubi, Archipelayo and Egg Toss); gamification features such as anonymous high score boards and achievements are available. Games were designed to encourage the player to blow in the prescribed breath length and pressure range (slightly extended expiration, 15-20cmH_2O), typically in multiple cycles of 8-10 breath sets with pauses for cough/huff. The number of breaths and cycles in the game is able to be altered by the player, reflecting the personalised ACT prescriptions of children in the study. Gaming is optional for participants during ACTs, but whether games have been played, or not, can be identified, including which games were played during specific sessions .

Participant questionnaires

Physiotherapy prescription including: ACT device(s), number of breaths and sets per day, estimated duration of ACT, exercise prescription and self-reported routine physical activities for individuals are collected by participant questionnaires at recruitment, mid-point and study end. An age specific CF questionnaire (CFQ-R: 6 to 11years, 12 to 13years or 14years+) is also administered at these timepoints for all participants. An additional parental CFQ-R is completed for those under 14years of age. The CFQ-R is a CF-specific measures of quality of life, with domains including respiratory and gastrointestinal symptoms, treatment burden and daily functioning for people with CF; it is currently the best validated CF-specific patient reported outcome measure,[10].

Clinical data

GOSH clinical data are extracted from electronic patient records (EPIC Systems Corp, Wisconsin, USA), pseudonymised and uploaded directly to the DRE. Historical data from older patient record systems (that is not available in EPIC) will be extracted, pseudonymised and uploaded to the DRE separately. This dataset contains as far as possible a participant's entire clinical record including known key features for CF recorded in the UK CF registry,[11] such as: genotype, height, weight, medications, hospital admissions, lung function, pancreatic status, microbiology, co-morbidities, ACT prescription and exercise test results.

For peer review only

At RLH and RBH electronic patient records are not currently available. Instead, clinical information will be extracted from clinical records/databases and from data uploaded to the UK CF registry. Data from participating hospitals are pseudonymised at source, and transferred by secure file transfer protocol (sFTP) to the DRE.

A clinical data processing pipeline will be developed to summarise features describing patient demographics, phenotypes and clinical status as frequencies (e.g. number of hospital admissions) or over a set time (e.g. number of hospital admissions per year).

DATA ANALYSIS

Adherence to physiotherapy

The primary outcome measures are adherence to a participant's daily ACT prescription (during different study phases: Standard, Feedback, Gaming), and adherence to daily physical activity recommendations. ACT sensor (breath pressure) and Fitbit (heart rate, footsteps) data are processed via a data pipeline in R (R Foundation, Vienna, Austria) within the DRE to describe these outcomes. Clinical data outcomes will be used to investigate any relationship between physiotherapy adherence behaviours to specific clinical profiles.

Adherence to a medical treatment is the extent to which a patient follows the recommendations agreed upon with health professionals,[12]. As physiotherapists give personalised ACT prescriptions, of varying breath count, set count and treatment session number per day, personalised adherence scores will be calculated. Daily ACT adherence will be quantified by the number and type of breaths recorded in a treatment session and per day. Cumulative, aggregate and week average scores will also be calculated. Adherence to the prescribed breath count (proportion of completed breaths against prescribed breaths per treatment (usually around 100 breaths)), prescribed breath pressures (proportion of ideal breath pressure breaths (15-20cmH_2O) per treatment) and prescribed breath length (proportion of expiratory breaths at ideal length (at least 1-2seconds) and also the presence of gaps for huff and cough between sets will be assessed. A composite adherence score based on these parameters combined per session, per day and per week will be calculated.

WHO guidelines recommend that all "*children and youth aged 5-17years should accumulate at least 60minutes of MVPA daily*"[13]. Adherence will be measured against this target for all participants. Time in MVPA is calculated daily as time where heart rate is greater than a personalised threshold value (usually around 120 beats per minute). A number of published approaches,[14] are being investigated to determine the optimal heart rate cut off value as no clear approach exists in the literature for the study population.

Data Processing Pipeline

A data processing pipeline enabling large amounts of remotely monitored sensor data to be processed efficiently, reliably, and reproducibly was developed in collaboration with Microsoft computer and data science engineers. It was developed using preliminary data collected in the first three months of data collection (September to December 2018) and then tested on a larger dataset (to April 2019).

The pipeline ensures data is processed consistently and data validation checks at each stage alert to errors. The pipeline is being refined throughout the ongoing data collection period, but briefly, it processes data through 3 main steps: *data cleaning* to remove errors, *data labelling* to mark and measure predefined constructs from raw data (for example distinct breaths from pressure traces)

For peer review only

and finally *data featurisation*, the quantification of variables for cluster analysis. These steps and the main features for analysis are summarised in table 2.

Statistical analysis

The large amount of heterogeneous data to be recorded and collected for each participant will be analysed using R and visualized using R Shiny apps. Any changes in adherence to physiotherapy prescriptions or recommendations over time or in relation to ACT gaming or feedback, will be quantified. As CF phenotypes and physiotherapy adherence are complex and multifactorial, cluster analysis will define groups of individuals based on measured characteristics to identify subgroups of participants with distinct physical activity and/or ACT adherence profiles.

The Fitbit and ACT sensor dataset currently contains 50+ variables (key features shown in table 2), with more to be added including from ongoing clinical record extraction. A correlation analysis of the features describing physiotherapy behaviours will remove features which are highly correlated. Variables with a Gaussian distribution will be normalised. Dimensionality reduction will be performed via principal component analysis. These methods will identify the most relevant and independent variables for cluster analysis to ensure a robust definition and visualisation of clusters.

Multiple features of ACT treatment sessions or activity days identified from the principal component and correlation analyses will be grouped using unsupervised cluster analysis, such that both the within-cluster similarity and between-cluster dissimilarity are strong. Unsupervised cluster analysis with the k-means method,[15] is a popular clustering technique and involves grouping multifactorial data into a pre-specified number of clusters. Numerous rapid computations comparing the relationship between multiple variables will determine the optimal cluster centres with the best separation between groups. The silhouette metric will be used to assess cluster homogeneity and separation, to identify the optimal number of clusters.

The associations, patterns and trends of individual physiotherapy adherence clusters to health outcomes (e.g. lung function, number of hospital admissions, infections, antibiotics etc) will be investigated with data from clinical records. Individual participant changes between clusters over time or in relation to gaming or feedback will also be investigated.

For peer review only

Table 2: Data pipeline stages and features for cluster analysis

**Sensor**<br>Type of data	**Data pipeline step**			
	**Cleaning**	**Labelling (of)**	**Featurisation** of variables for cluster analysis	
			Descriptive	Describing Adherence$^+$
**ACT Sensor**<br>Pressure-Time	• Removal of blank, duplicate and non-physiological values.<br>• Any non-linear baseline drift corrected, using a sparsity based de-noising approach,[16].	• Treatment sessions<br>• Pressure peaks<br>• Breaths<br>• Breaks between breaths<br>• Sets of breaths	• If any breaths recorded on a day Y/N<br>• Breath count*<br>• Breath length*<br>• Breath peak pressure*<br>• Treatment duration*<br>• Number of treatments per day<br>• Number of sets in a treatment *<br>• Number of breaths per set*	• Adherence score (proportion of days with any breaths recorded per total number of days)<br>• Breath count adherence (proportion of completed breaths against prescribed breaths per treatment)<br>• Set adherence (proportion of sets against prescribed sets per treatment)<br>• Treatment session adherence (proportion of completed treatments against prescribed treatments)<br>• Pressure adherence (proportion of ideal expiratory pressure breaths per treatment)<br>• Breath length adherence (proportion of expiratory breaths at ideal length per treatment)
**Fitbit**<br>Heart rate and footsteps	• Removal of erroneous or non-physiological data (caused by improper wearing, depleted battery, full chip memory capacity due to infrequent syncing).<br>• Heart rate sampling frequency made consistent (per minute) using a rolling average	• Gaps in data<br>• Wear time (from heart rate data)<br>• Awake wear time<br>• Time in MVPA using personalised heart rate cut off value [14]<br>• Points crossing MVPA cut off value	• Heart rate<br>○ Resting[17]<br>○ Peak<br>○ Density and variability<br>○ MVPA threshold switches<br>• Footsteps<br>○ Daily step count<br>○ Density and variability<br>○ Active minutes >threshold value<br>• Combined<br>○ Active minutes both heart rate and footstep.<br>○ Step count during periods of MVPA	• Daily time in MVPA compared to 60 daily minutes recommended.<br>• Weekly time in MVPA, 7-day window compared to 420 weekly minutes recommended.

Not all features shown. $^+$ Features quantified as adherence per prescribed treatment session, per day, per rolling 7 day week, or other time point as required for analysis. ACT prescription taken from clinical records and physiotherapy questionnaires. * Total and/or average per treatment, standard deviation, min, max values

For peer review only - http://bmjopen.bmj.com/site/about/guidelines.xhtml

Dissemination

Study findings will be disseminated via peer-reviewed publications in open source and high impact journals, relevant national and international conferences and via CF clinical and patient networks. An ACTNow Award (October 2019) means high quality resources to disseminate results to both the CF population and participants in the study will be produced and results published on our website https://fizzyo.github.io/. The statistical code, will be published in the Fizzyo GitHub repository and the dataset stored and available through the GOSH DRE.

Author contributions

EM, MB, HD, HS, GS, LS conceived and designed the study. GS perceived and developed the sensor and with LS and TK the data processing infrastructure including the bespoke app. EM, ER, HD developed the protocol with input from all investigators. ER, HD, NM, RC recruited and support all participants. JB is the DRE data steward and facilitator of electronic patient record extraction. KK, NF, OL, TVS, BF developed the data processing pipeline and preliminary analysis with EM, GD, NM, RC, HD, MB providing clinical expertise for featurisation. ER, KK, EM, NF, HD will conduct the data analyses. ER and EM wrote the first draft of the manuscript, all authors reviewed this and approved the final manuscript.

Acknowledgements

With thanks to the clinical CF Teams at each of the recruiting sites, the GOSH DRE team and Microsoft UK, especially Haiyan Zhang, Simon Jackson, and the Microsoft CSE including Josh Lane, Pete Roden, Stephanie Marker, Christian Robles, Kristjana Popovski, Hannah Kennedy and Kristin Ottofy. Also Ryan White, Michael Woollard and Alan Bannon for electronic engineering input, Dean Mohamedally and UCL students (computer science), and Jamie Bankhead and the team at Abertay University for gaming designs and UCL physiotherapy MSc students.

Funding

This work was supported by the UCL Rosetrees Stoneygate prize (M712), a Cystic Fibrosis Trust Clinical Excellence and Innovation Award (CEA010), A UCL Partners award and the HEFCE Higher Education Innovation Fund (KEI2017-01-04). All work at UCL GOSICH is supported by the NIHR GOSH BRC. The views expressed are those of the author(s) and not necessarily those of the NHS, the NIHR or the Department of Health. The study is sponsored by University College London. The funders and sponsor played no role in the design of the study.

Conflicts of interest

The authors declare that they have no conflicts of interest in relation to this protocol.

Protocol registration

This study was approved by the London- Brighton and Sussex, NHS Research Ethics Committee (18/LO/1038) and it is registered with ISRCTN (51624752). We used the SPIRIT checklist when writing our report,[18].

For peer review only

References

1. Smyth AR, Bell SC, Bojcin S, et al. European Cystic Fibrosis Society Standards of Care: Best Practice guidelines. J Cyst Fibros. 2014;13 Suppl 1:S23-42.

2. Van der Schans CP, Prasad A, Main E. Chest physiotherapy compared to no chest physiotherapy for cystic fibrosis. Cochrane Database of Systematic Reviews. 2000(2).

3. Davies G, Rowbotham NJ, Smith S, et al. Characterising burden of treatment in cystic fibrosis to identify priority areas for clinical trials. Journal of Cystic Fibrosis. 2019.

4. James Lind Alliance Priority Setting Partnership. Cystic Fibrosis Top 10 Priorities [Webpage]. 2017 [Available from: http://www.jla.nihr.ac.uk/priority-setting-partnerships/cystic-fibrosis/top-10-priorities.htm.

5. Mikesell CL, Kempainen RR, Laguna TA, et al. Objective Measurement of Adherence to Out-Patient Airway Clearance Therapy by High-Frequency Chest Wall Compression in Cystic Fibrosis. 2017;62(7):920-7.

6. Main E. What is the best airway clearance technique in cystic fibrosis? Paediatric Respiratory Reviews. 2013;14:10-2.

7. Wilson LM, Morrison L, Robinson KA. Airway clearance techniques for cystic fibrosis: an overview of Cochrane systematic reviews. Cochrane Database of Systematic Reviews. 2019(1).

8. Schneiderman JE, Wilkes DL, Atenafu EG, et al. Longitudinal relationship between physical activity and lung health in patients with cystic fibrosis. European Respiratory Journal. 2014;43(3):817-23.

9. Bradley JM, Moran FM, Stuart Elborn J. Evidence for physical therapies (airway clearance and physical training) in cystic fibrosis: An overview of five Cochrane systematic reviews. Respir Med. 2006;100(2):191-201.

10. Ronit A, Gelpi M, Argentiero J, et al. Electronic applications for the CFQ-R scoring. Respiratory Research. 2017;18(1):108.

11. Registry UCF. UK Cystic Fibrosis Registry Annual Data Report 2018 - Cystic Fibrosis Trust. 2019.

12. World Health Organization, Adherence to long term therapies, Evidence for action: World Health Organization; 2003 [Available from: https://www.who.int/chp/knowledge/publications/adherence_report/en/.

13. World Health Organization, Physical activity and young people 2020 [Available from: https://www.who.int/dietphysicalactivity/factsheet_young_people/en/.

14. Douglas H, Raywood E, Kapoor K, et al. ePS3.06 Quantifying moderate to vigorous physical activity using heart rate in children and young people with cystic fibrosis. Journal of Cystic Fibrosis. 2019;18:S46.

15. Ding C, He X. K-means clustering via principal component analysis. Proceedings of the twenty-first international conference on Machine learning; Banff, Alberta, Canada: Association for Computing Machinery; 2004. p. 29.

16. Ning X, Selesnick IW, Duval L. Chromatogram baseline estimation and denoising using sparsity (BEADS). Chemometrics and Intelligent Laboratory Systems. 2014;139:156-67.

17. Kapoor K, Raywood E, Douglas H, et al. Determining resting heart rate in children using wearable activity monitors. European Respiratory Journal. 2019;54(suppl 63):PA342.

18. Chan AW, Tetzlaff JM, Altman DG, et al. SPIRIT 2013 statement: defining standard protocol items for clinical trials. Ann Intern Med. 2013;158(3):200-7.

Figure Legends

Figure 1: Study design flowchart of the 16month study per participant. Participant visits for assessments are in shaded boxes. Airway clearance techniques (ACTs) and physical activity are

For peer review only

recorded daily using electronically chipped devices (a bespoke ACT sensor and a Fitbit activity tracker). The specially developed Fizzyo app as well as for syncing ACT data throughout the 16 months, gives participant feedback in months 2-14 and ACT-driven gaming is available in months 4-12.

Figure 2: Project Fizzyo data collection pathway. Remote monitoring sensors (Fizzyo sensor, Fitbit) connect via Bluetooth to sync data with device specific apps on a study tablet. Anonymous data is sent to the Fizzyo cloud (either directly or via application programming interface from the Fitbit cloud) and then linked with de-identified clinical records in the GOSH Digital Research Environment.

Figure 3: Fizzyo sensor and connectors attached to an Acapella® Choice ACT device. Breathing through the mouthpiece changes flow and pressure within the ACT device which is recorded by the sensor.

For peer review only

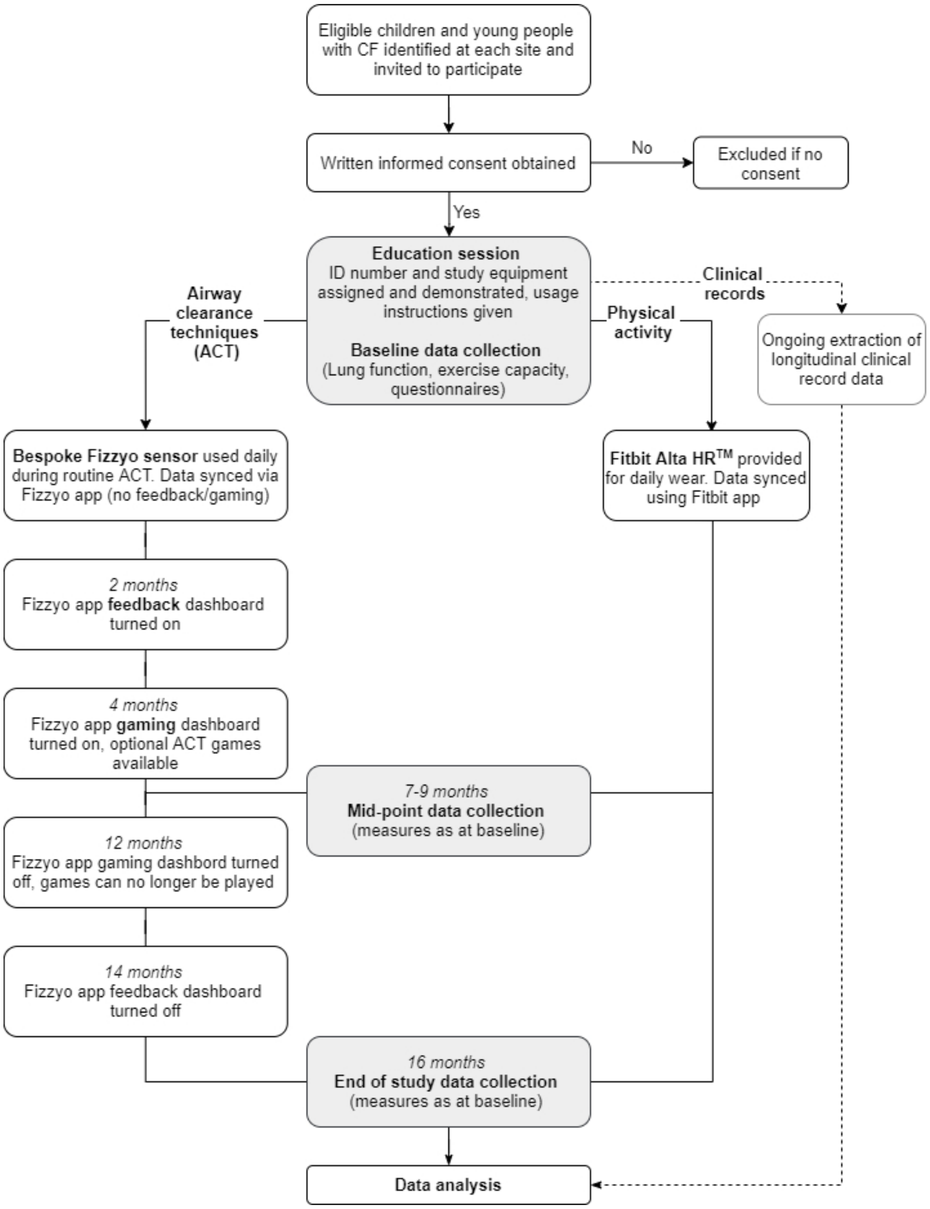

Figure 1: Study design flowchart of the 16month study per participant. Participant visits for assessments are in shaded boxes. Airway clearance techniques (ACTs) and physical activity are recorded daily using electronically chipped devices (a bespoke ACT sensor and a Fitbit activity tracker). The specially developed Fizzyo app as well as for syncing ACT data throughout the 16 months, gives participant feedback in months 2-14 and ACT-driven gaming is available in months 4-12.

252x328mm (72 x 72 DPI)

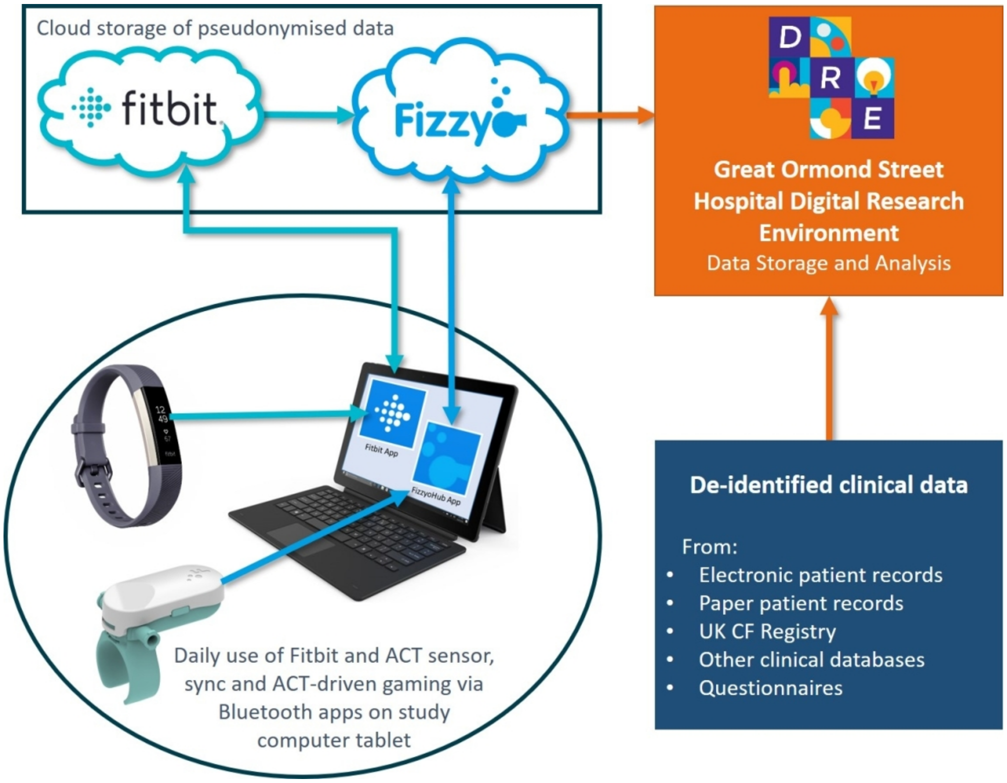

Figure 2: Project Fizzyo data collection pathway. Remote monitoring sensors (Fizzyo sensor, Fitbit) connect via Bluetooth to sync data with device specific apps on a study tablet. Anonymous data is sent to the Fizzyo cloud (either directly or via application programming interface from the Fitbit cloud) and then linked with de-identified clinical records in the GOSH Digital Research Environment.

240x185mm (150 x 150 DPI)

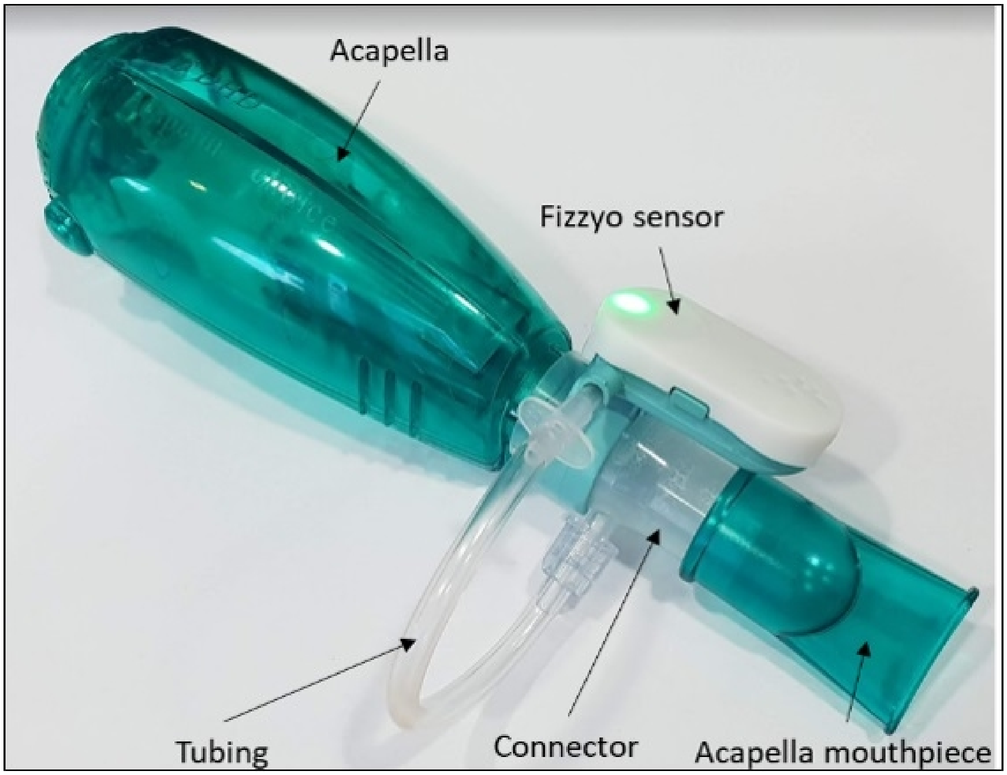

Figure 3: Fizzyo sensor and connectors attached to an Acapella® Choice ACT device. Breathing through the mouthpiece changes flow and pressure within the ACT device which is recorded by the sensor.

150x115mm (96 x 96 DPI)

Reporting checklist for protocol of a clinical trial.

Based on the SPIRIT guidelines.

Instructions to authors

Complete this checklist by entering the page numbers from your manuscript where readers will find each of the items listed below.

Your article may not currently address all the items on the checklist. Please modify your text to include the missing information. If you are certain that an item does not apply, please write "n/a" and provide a short explanation.

Upload your completed checklist as an extra file when you submit to a journal.

In your methods section, say that you used the SPIRITreporting guidelines, and cite them as:

Chan A-W, Tetzlaff JM, Altman DG, Laupacis A, Gøtzsche PC, Krleža-Jerić K, Hróbjartsson A, Mann H, Dickersin K, Berlin J, Doré C, Parulekar W, Summerskill W, Groves T, Schulz K, Sox H, Rockhold FW, Rennie D, Moher D. SPIRIT 2013 Statement: Defining standard protocol items for clinical trials. Ann Intern Med. 2013;158(3):200-207

		Reporting Item	Page Number
**Administrative information**			
Title	#1	Descriptive title identifying the study design, population, interventions, and, if applicable, trial acronym	1
Trial registration	#2a	Trial identifier and registry name. If not yet registered, name of intended registry	1
Trial registration: data set	#2b	All items from the World Health Organization Trial Registration Data Set	n/a
Protocol version	#3	Date and version identifier	2
Funding	#4	Sources and types of financial, material, and other support	11
Roles and responsibilities: contributorship	#5a	Names, affiliations, and roles of protocol contributors	1, 11

For peer review only

Roles and responsibilities: sponsor contact information	#5b	Name and contact information for the trial sponsor	n/a
Roles and responsibilities: sponsor and funder	#5c	Role of study sponsor and funders, if any, in study design; collection, management, analysis, and interpretation of data; writing of the report; and the decision to submit the report for publication, including whether they will have ultimate authority over any of these activities	11
Roles and responsibilities: committees	#5d	Composition, roles, and responsibilities of the coordinating centre, steering committee, endpoint adjudication committee, data management team, and other individuals or groups overseeing the trial, if applicable (see Item 21a for data monitoring committee)	n/a
**Introduction**			
Background and rationale	#6a	Description of research question and justification for undertaking the trial, including summary of relevant studies (published and unpublished) examining benefits and harms for each intervention	3
Background and rationale: choice of comparators	#6b	Explanation for choice of comparators	8
Objectives	#7	Specific objectives or hypotheses	n/a
Trial design	#8	Description of trial design including type of trial (eg, parallel group, crossover, factorial, single group), allocation ratio, and framework (eg, superiority, equivalence, non-inferiority, exploratory)	4
**Methods: Participants, interventions, and outcomes**			
Study setting	#9	Description of study settings (eg, community clinic, academic hospital) and list of countries where data will be collected. Reference to where list of study sites can be	4

For peer review only

		obtained	
Eligibility criteria	#10	Inclusion and exclusion criteria for participants. If applicable, eligibility criteria for study centres and individuals who will perform the interventions (eg, surgeons, psychotherapists)	4
Interventions: description	#11a	Interventions for each group with sufficient detail to allow replication, including how and when they will be administered	4
Interventions: modifications	#11b	Criteria for discontinuing or modifying allocated interventions for a given trial participant (eg, drug dose change in response to harms, participant request, or improving / worsening disease)	n/a
Interventions: adherance	#11c	Strategies to improve adherence to intervention protocols, and any procedures for monitoring adherence (eg, drug tablet return; laboratory tests)	4
Interventions: concomitant care	#11d	Relevant concomitant care and interventions that are permitted or prohibited during the trial	n/a
Outcomes	#12	Primary, secondary, and other outcomes, including the specific measurement variable (eg, systolic blood pressure), analysis metric (eg, change from baseline, final value, time to event), method of aggregation (eg, median, proportion), and time point for each outcome. Explanation of the clinical relevance of chosen efficacy and harm outcomes is strongly recommended	6-8, table 2
Participant timeline	#13	Time schedule of enrolment, interventions (including any run-ins and washouts), assessments, and visits for participants. A schematic diagram is highly recommended (see Figure)	figure 1
Sample size	#14	Estimated number of participants needed to achieve study objectives and how it was determined, including clinical and statistical assumptions supporting any sample size calculations	4
Recruitment	#15	Strategies for achieving adequate participant enrolment to reach target sample size	4

For peer review only

Methods: Assignment of interventions (for controlled trials)

Allocation: sequence generation	#16a	Method of generating the allocation sequence (eg, computer-generated random numbers), and list of any factors for stratification. To reduce predictability of a random sequence, details of any planned restriction (eg, blocking) should be provided in a separate document that is unavailable to those who enrol participants or assign interventions	n/a
Allocation concealment mechanism	#16b	Mechanism of implementing the allocation sequence (eg, central telephone; sequentially numbered, opaque, sealed envelopes), describing any steps to conceal the sequence until interventions are assigned	n/a
Allocation: implementation	#16c	Who will generate the allocation sequence, who will enrol participants, and who will assign participants to interventions	n/a
Blinding (masking)	#17a	Who will be blinded after assignment to interventions (eg, trial participants, care providers, outcome assessors, data analysts), and how	n/a
Blinding (masking): emergency unblinding	#17b	If blinded, circumstances under which unblinding is permissible, and procedure for revealing a participant's allocated intervention during the trial	n/a

Methods: Data collection, management, and analysis

Data collection plan	#18a	Plans for assessment and collection of outcome, baseline, and other trial data, including any related processes to promote data quality (eg, duplicate measurements, training of assessors) and a description of study instruments (eg, questionnaires, laboratory tests) along with their reliability and validity, if known. Reference to where data collection forms can be found, if not in the	5-7

For peer review only

		protocol	
Data collection plan: retention	#18b	Plans to promote participant retention and complete follow-up, including list of any outcome data to be collected for participants who discontinue or deviate from intervention protocols	n/a
Data management	#19	Plans for data entry, coding, security, and storage, including any related processes to promote data quality (eg, double data entry; range checks for data values). Reference to where details of data management procedures can be found, if not in the protocol	6-7
Statistics: outcomes	#20a	Statistical methods for analysing primary and secondary outcomes. Reference to where other details of the statistical analysis plan can be found, if not in the protocol	8-9
Statistics: additional analyses	#20b	Methods for any additional analyses (eg, subgroup and adjusted analyses)	8-9
Statistics: analysis population and missing data	#20c	Definition of analysis population relating to protocol non-adherence (eg, as randomised analysis), and any statistical methods to handle missing data (eg, multiple imputation)	n/a
**Methods: Monitoring**			
Data monitoring: formal committee	#21a	Composition of data monitoring committee (DMC); summary of its role and reporting structure; statement of whether it is independent from the sponsor and competing interests; and reference to where further details about its charter can be found, if not in the protocol. Alternatively, an explanation of why a DMC is not needed	n/a
Data monitoring: interim analysis	#21b	Description of any interim analyses and stopping guidelines, including who will have access to these interim results and make the final decision to terminate the trial	n/a
Harms	#22	Plans for collecting, assessing, reporting, and managing solicited and spontaneously reported adverse events and other unintended effects of trial interventions or trial conduct	n/a
Auditing	#23	Frequency and procedures for auditing trial conduct, if	n/a

For peer review only

		any, and whether the process will be independent from investigators and the sponsor	
**Ethics and dissemination**			
Research ethics approval	#24	Plans for seeking research ethics committee / institutional review board (REC / IRB) approval	2
Protocol amendments	#25	Plans for communicating important protocol modifications (eg, changes to eligibility criteria, outcomes, analyses) to relevant parties (eg, investigators, REC / IRBs, trial participants, trial registries, journals, regulators)	n/a
Consent or assent	#26a	Who will obtain informed consent or assent from potential trial participants or authorised surrogates, and how (see Item 32)	5
Consent or assent: ancillary studies	#26b	Additional consent provisions for collection and use of participant data and biological specimens in ancillary studies, if applicable	n/a
Confidentiality	#27	How personal information about potential and enrolled participants will be collected, shared, and maintained in order to protect confidentiality before, during, and after the trial	6
Declaration of interests	#28	Financial and other competing interests for principal investigators for the overall trial and each study site	11
Data access	#29	Statement of who will have access to the final trial dataset, and disclosure of contractual agreements that limit such access for investigators	11
Ancillary and post trial care	#30	Provisions, if any, for ancillary and post-trial care, and for compensation to those who suffer harm from trial participation	4
Dissemination policy: trial results	#31a	Plans for investigators and sponsor to communicate trial results to participants, healthcare professionals, the public, and other relevant groups (eg, via publication, reporting in results databases, or other data sharing arrangements), including any publication restrictions	11
Dissemination policy:	#31b	Authorship eligibility guidelines and any intended use of	n/a

For peer review only

authorship		professional writers	
Dissemination policy: reproducible research	#31c	Plans, if any, for granting public access to the full protocol, participant-level dataset, and statistical code	11
**Appendices**			
Informed consent materials	#32	Model consent form and other related documentation given to participants and authorised surrogates	n/a
Biological specimens	#33	Plans for collection, laboratory evaluation, and storage of biological specimens for genetic or molecular analysis in the current trial and for future use in ancillary studies, if applicable	n/a

Notes:

- 12: 6-8, table 2 The SPIRIT checklist is distributed under the terms of the Creative Commons Attribution License CC-BY-ND 3.0. This checklist was completed on 15. April 2020 using https://www.goodreports.org/, a tool made by the EQUATOR Network in collaboration with Penelope.ai

For peer review only

BMJ Open

Protocol for Project Fizzyo, an analytic longitudinal observational cohort study of physiotherapy for children and young people with cystic fibrosis, with interrupted time series design

Journal:	*BMJ Open*
Manuscript ID	bmjopen-2020-039587.R1
Article Type:	Protocol
Date Submitted by the Author:	15-Jul-2020
Complete List of Authors:	Raywood, Emma; University College London Institute of Child Health, Physiotherapy, Respiratory, Critical Care and Anaesthesia Section,<br>Douglas, Helen; University College London Institute of Child Health, Physiotherapy, Respiratory, Critical Care and Anaesthesia Section,<br>Kapoor, Kunal; University College London Institute of Child Health, Physiotherapy, Respiratory, Critical Care and Anaesthesia Section,<br>Filipow, Nicole; University College London Institute of Child Health, Physiotherapy, Respiratory, Critical Care and Anaesthesia Section,<br>Murray, Nicky; Royal Brompton and Harefield NHS Foundation Trust, Paediatric Cystic Fibrosis Unit,<br>O'Connor, Rachel; Barts Health NHS Trust, Paediatric Cystic Fibrosis Centre, Royal London Hospital,<br>Stott, Lee; Microsoft UK Ltd - Reading, Commercial Software Engineering,<br>Saul, Greg; Microsoft Research Ltd, Microsoft Research Lab,<br>Kuzhagaliyev, Tim; University College London Department of Computer Science, Computer Science<br>Davies, Gwyneth ; University College London Institute of Child Health, Respiratory Critical Care and Anaesthesia section,<br>Liakhovich, Olga; Microsoft UK Ltd - Reading, Commercial Software Engineering,<br>Van Schaik, Tempest; Microsoft UK Ltd - Reading, Commercial Software Engineering,<br>Furtuna, Bianca; Microsoft UK Ltd - Reading, Commercial Software Engineering,<br>Booth, John; Great Ormond Street Hospital For Children NHS Foundation Trust, Digital Research Environment,<br>Shannon, Harriet; University College London Institute of Child Health, Physiotherapy, Respiratory, Critical Care and Anaesthesia Section,<br>Bryon, Mandy; Great Ormond Street Hospital For Children NHS Foundation Trust, Department of Paediatric Psychology,<br>Main, Eleanor; University College London Institute of Child Health, Physiotherapy, Respiratory, Critical Care and Anaesthesia Section,
**Primary Subject Heading**:	Respiratory medicine
Secondary Subject Heading:	Paediatrics, Health informatics, Research methods

Keywords:	Cystic fibrosis < THORACIC MEDICINE, Paediatric thoracic medicine < PAEDIATRICS, BIOTECHNOLOGY & BIOINFORMATICS, Protocols & guidelines < HEALTH SERVICES ADMINISTRATION & MANAGEMENT

SCHOLARONE™
Manuscripts

I, the Submitting Author has the right to grant and does grant on behalf of all authors of the Work (as defined in the below author licence), an exclusive licence and/or a non-exclusive licence for contributions from authors who are: i) UK Crown employees; ii) where BMJ has agreed a CC-BY licence shall apply, and/or iii) in accordance with the terms applicable for US Federal Government officers or employees acting as part of their official duties; on a worldwide, perpetual, irrevocable, royalty-free basis to BMJ Publishing Group Ltd ("BMJ") its licensees and where the relevant Journal is co-owned by BMJ to the co-owners of the Journal, to publish the Work in this journal and any other BMJ products and to exploit all rights, as set out in our licence.

The Submitting Author accepts and understands that any supply made under these terms is made by BMJ to the Submitting Author unless you are acting as an employee on behalf of your employer or a postgraduate student of an affiliated institution which is paying any applicable article publishing charge ("APC") for Open Access articles. Where the Submitting Author wishes to make the Work available on an Open Access basis (and intends to pay the relevant APC), the terms of reuse of such Open Access shall be governed by a Creative Commons licence – details of these licences and which Creative Commons licence will apply to this Work are set out in our licence referred to above.

Other than as permitted in any relevant BMJ Author's Self Archiving Policies, I confirm this Work has not been accepted for publication elsewhere, is not being considered for publication elsewhere and does not duplicate material already published. I confirm all authors consent to publication of this Work and authorise the granting of this licence.

For peer review only

bmjopen-2020-039587.R1

Protocol for Project Fizzyo, an analytic longitudinal observational cohort study of physiotherapy for children and young people with cystic fibrosis, with interrupted time series design

Authors: Emma Raywood[1] e.raywood@ucl.ac.uk, Helen Douglas[1] h.douglas@ucl.ac.uk, Kunal Kapoor[1] k.kapoor@ucl.ac.uk, Nicole Filipow[1] nicole.filipow.18@ucl.ac.uk, Nicky Murray[2] nickymurray@nhs.net, Rachel O'Connor[3] rachel.oconnor1@nhs.net, Lee Stott[4] leestott@microsoft.com, Greg Saul[5] gregsaul@gmail.com, Tim Kuzhagaliyev[6] tim.kuzh@gmail.com, Gwyneth Davies[1] gwyneth.davies@ucl.ac.uk, Olga Liakhovich[4] olga.liakhovich@microsoft.com, Tempest Van Schaik[4] tempest.van@microsoft.com, Bianca Furtuna[4] bianca.furtuna@microsoft.com, John Booth[7] john.booth@gosh.nhs.uk, Harriet Shannon[1] h.shannon@ucl.ac.uk, Mandy Bryon[8] mandy.bryon@gosh.nhs.uk, and Eleanor Main[1] e.main@ucl.ac.uk.

Affiliations: [1] Physiotherapy, Respiratory Critical Care and Anaesthesia section, UCL Great Ormond Street Institute of Child Health, London, UK, [2] Paediatric Cystic Fibrosis Unit, Royal Brompton Hospital, Royal Brompton & Harefield NHS Foundation Trust, London, UK, [3] Paediatric Cystic Fibrosis Centre, Royal London Hospital, Barts Health NHS Trust, London, UK, [4] Commercial Software Engineering, Microsoft UK, London/Reading UK, [5] Microsoft Research Lab, Microsoft Research, Cambridge, UK, [6] Department of Computer Science, UCL, London, UK. [7] DRIVE, Great Ormond Street Hospital for Children NHS Foundation Trust, London, UK, [8] Department of Paediatric Psychology, Great Ormond Street Hospital for Children NHS Foundation Trust, London, UK,

Corresponding Author: Emma Raywood, e.raywood@ucl.ac.uk Physiotherapy, Respiratory Critical Care and Anaesthesia section, UCL Great Ormond Street Institute of Child Health, London, UK.

Keywords: Cystic fibrosis, Data science, Paediatrics, Physical activity, Physiotherapy

Word Count: 3985 words

ABSTRACT

Introduction: Daily physiotherapy is believed to mitigate the progression of cystic fibrosis (CF) lung disease. However, physiotherapy airway clearance techniques (ACTs) are burdensome and the evidence guiding practice remains weak. This paper describes the protocol for Project Fizzyo, which uses innovative technology and analysis methods to remotely capture longitudinal daily data from physiotherapy treatments to measure adherence and prospectively evaluate associations with clinical outcomes.

Methods and analysis: A cohort of 145 children and young people with CF aged 6-16 years were recruited. Each participant will record their usual physiotherapy sessions daily for 16 months, using remote monitoring sensors: 1) a bespoke ACT sensor, inserted into their usual ACT device and 2) a Fitbit Alta HR™ activity tracker. Real time breath pressure during ACTs, and heart rate and daily step counts (Fitbit) are synced using specific software applications. An interrupted time series design will facilitate evaluation of ACT interventions (feedback and ACT-driven gaming).

Baseline, mid and end-point assessments of spirometry, exercise capacity, and quality of life and longitudinal clinical record data will also be collected.

For peer review only - http://bmjopen.bmj.com/site/about/guidelines.xhtml

This large dataset will be analysed in R using big data analytics approaches. Distinct ACT and physical activity adherence profiles will be identified using cluster analysis to define groups of individuals based on measured characteristics and any relationships to clinical profiles assessed. Changes in adherence to physiotherapy over time or in relation to ACT interventions will be quantified and evaluated in relation to clinical outcomes.

Ethics and dissemination: Ethical approval for this study (IRAS: 228625) was granted by the London-Brighton and Sussex NREC (18/LO/1038). Findings will be disseminated via peer-reviewed publications, at conferences and via CF clinical networks. The statistical code will be published in the Fizzyo GitHub repository and the dataset stored and available through the Great Ormond Street Hospital Digital Research Environment.

Registration: ISRCTN51624752

ARTICLE SUMMARY

Strengths and limitations of this study

- This research is directly related to four of the James Lind Alliance top ten research priorities for CF; simplifying treatment burden, evidence for effectiveness of therapies to delay the onset of lung disease in early life, the application of new technology and exercise as a potential replacement for ACT.
- The longitudinal observational study design and novel use of daily remote monitoring captures detailed objective data from usual physiotherapy routine care, and has the potential to provide real-world evidence to guide ACT practice where randomised controlled trials have failed.
- Variability in synchronisation or use of the ACT sensor and Fitbit or intermittent technical failure of either will make analysis of remotely collected adherence data complex and challenging.
- Budget constraints, patient preference and the requirement to extract raw heart rate and step count data limited the choice of activity tracker which represented a potential compromise between data quality and willingness to wear the tracker.
- The development of a sustainable big data infrastructure including a pipeline for recording, syncing, processing and analysis of remote monitoring and clinical data was integral to this study and is vital for the future use of these technologies.

For peer review only

For peer review only - http://bmjopen.bmj.com/site/about/guidelines.xhtml

INTRODUCTION

Cystic Fibrosis (CF) is a life-limiting inherited condition, which affects over 10,000 people in the UK. Despite recent treatment advances, including modulator therapies that target the underlying cause of the disease,[1] CF remains progressive and incurable. People with CF are susceptible to repeated respiratory infections due to thick respiratory mucus, which can lead to irreversible lung damage. Daily treatment to slow the progression of lung disease, and therefore the onset of respiratory failure, is the primary aim of almost all current CF therapies,[2]. Daily physiotherapy, including airway clearance techniques (ACTs), physical activity and exercise, is believed to mitigate the progression of CF lung disease,[3].

People with CF undertake a median of ten different concurrent treatments that take an average of two hours each day,[4]. This high daily treatment burden (and high cost of care) from childhood has become a driver for research to maximise effective care and minimise unnecessary therapies. Reducing treatment burden has been identified as the top CF research priority by the James Lind Alliance,[5]. Follow-up research found that although ACTs were perceived as very important, they were considered the most burdensome daily treatment,[4]. ACTs are stressful for people with CF and their families, and adherence can be low. Of those questioned, 70% said they regularly missed some of their daily treatments, most commonly avoiding ACTs or nebulised therapies,[4]. Another study found those with the lowest adherence to chest physiotherapy had worse lung function, more exacerbations and consequently had higher health costs,[6].

Another of the James Lind Alliance top ten research priorities for CF is to identify the specific therapies that could delay and prevent progression of lung disease in early life,[5]. Despite over 70 years of ACTs in routine CF clinical practice, the evidence base to guide treatment remains weak. A number of challenges exist for researchers in the field; a plethora of physiotherapy ACTs and devices exist, providing a bewildering choice for therapists and patients, with the long-term effects of different devices, techniques or non-adherence being poorly understood. Traditional research methods (including randomised controlled trials) have failed to produce credible evidence for optimal ACT type, dose, frequency or duration,[7, 8]. Established solitary outcome measures (e.g. FEV_1) are insensitive to change in mild CF lung disease, and are not useful end points for physiotherapy clinical trials. Furthermore blinding is not possible, patient or practitioner preferences can confound results of trials and ethical concerns about the complete removal of ACTs persist,[3]. There is evidence that patients with the lowest self-reported physical activity levels have poorer health,[9] but the use of exercise as an ACT remains controversial,[10] despite the fact it is popular with patients,[5].

Advances in technology, including increased use of electronically chipped devices, electronic patient records and the growth of big data analytics, are providing fresh opportunities for physiotherapy research. These may facilitate clarity and certainty about effective therapies and help to reduce treatment burden. Big data techniques, including machine learning and unsupervised clustering, are useful for analysis of healthcare data, which is often complex, unstructured and from multiple sources. However, they are yet to be used to build credible evidence to guide physiotherapy for people with CF.

Project Fizzyo

This paper describes the protocol for Project Fizzyo (ISRCTN51624752), which uses innovative technology to capture detailed longitudinal data from children and young people with CF (CYPwCF) undertaking daily physiotherapy treatments. Real time breath pressure during ACTs are captured

For peer review only - http://bmjopen.bmj.com/site/about/guidelines.xhtml

daily, as well as heart rate and step count during physical activity. The interrupted time series study design facilitates evaluation of interventions such as ACT feedback and ACT-driven gaming.

The aims of Project Fizzyo are 1) to monitor and understand adherence to daily ACT prescriptions, 2) to evaluate associations between ACT adherence and clinical outcomes under different study conditions (including feedback and gaming), 3) to monitor and understand daily patterns of physical activity in relation to published recommendations, and evaluate any associations with clinical outcomes, and 4) to use big data analytics to seek out important data signals in relation to optimising ACT and exercise prescriptions for CYPwCF.

METHODS

Study design

Project Fizzyo is an analytic longitudinal observational cohort study with an interrupted time series design. Clinically prescribed ACT or exercise prescriptions for individual participants are continued as normal for the 16 months of the study. Each participant uses two remote monitoring sensors to record data daily. These are: 1) a bespoke Fizzyo ACT breath pressure sensor, inserted into their regular ACT device (Acapella Choice®, Aerobika®, AstraTech PEP® or Pari PEP™) and 2) a Fitbit Alta HR™ activity tracker (Fitbit Inc, San Francisco, USA) to record daily physical activity (heart rate and step count). Participants synchronise (sync) data using apps for each sensor, on a tablet computer provided for the study.

ACT feedback (daily number of breaths) and ACT-driven computer gaming are introduced and removed in an interrupted time series (figure 1), via the specially developed Fizzyo app. Baseline ACT patterns are recorded (during months 0-2: standard phase), then sequential introduction of ACT feedback (during months 2-14: feedback phase) and ACT-driven gaming (during months 4-12: gaming phase) will allow any effect on breathing patterns, adherence or clinical outcomes to be observed. The removal of gaming (at month 12) and feedback (at month 14) will facilitate observation of lasting, temporary or feedback/game dependent behaviour changes.

Patient and Public Involvement

This study addresses top research priorities identified by people with CF, their families, carers and clinical teams as part of the James Lind Alliance CF Priority Setting Partnership,[4]. The ACT sensor and gaming were developed as part of the BBC2 programme, “The big life fix” at the request of a family with two sons who have CF,[11]. This idea was developed further by the study team, which includes a parent of children with CF, physiotherapy specialists and product design specialists. Children from Great Ormond Street Hospital (GOSH) and their families advised on the choice of Fitbit, development of the ACT sensor (including the addition of lights to indicate breathing), customisability of the Fizzyo app and games, the study design and information materials. Dissemination of study results to both to participants in the study and the wider CF population is planned.

Participants

To be eligible, participants at recruitment were: 1) aged 6-16 years, 2) diagnosed with CF and under the care of a participating London paediatric CF centre (either GOSH, the Royal London Hospital (RLH) or the Royal Brompton Hospital (RBH), including shared care patients), and 3) prescribed one of the four ACT devices compatible with the ACT sensor, at least once a day as part of routine ACTs.

For peer review only - http://bmjopen.bmj.com/site/about/guidelines.xhtml

Patients were excluded if 1) they/their parents did not or could not provide informed consent, 2) they were not prescribed one of the 4 specific ACT devices as part of their routine daily treatment, (pre-study self reported non-adherence to prescribed ACT did not prevent children from participation) 3) had undergone lung transplantation or 4) had a clinically significant medical condition other than CF.

Children meeting the eligibility criteria were approached by their clinical team with the study information sheets. Participant information sheets affirm that no direct benefits are promised to participants, although they may find the computer games help them to enjoy and engage with airway clearance. After 16 months the study will end for each participant and the ACT sensor and Fitbit will be returned. Participants can choose to keep the devices, with restored games and feedback for a short period at the end of their participation, on the condition that they continue to sync data until the remaining period of data collection is concluded for all participants (December 2020).

Participants were recruited from September 2018 to July 2019. The recruitment window was scheduled to be open for 6 months per site, though for the first and largest recruiting site (GOSH) an extra month was added to allow for a period of pilot data collection to resolve early technical issues. The initial recruitment target of 160 participants was reduced to 145 due to manufacturing delays for the bespoke Fizzyo sensor, which was not a threat to meeting the aims of the study. This study, with 145 CYPwCF is the largest CF population sample in any physiotherapy study undertaken in Europe to date. Daily data from these children over 16 months will provide sufficient precision of estimation in evaluating the signals of adherence to therapy.

The number of children (recruitment periods) at each hospital were: at GOSH 75, (10/09/2018-16/04/2019), RBH 40, (30/11/2018-31/05/2019) and RLH 30, (03/01-01/07/2019). Data collection is ongoing and is predicted to end in December 2020.

Data collection

Informed consent to participate in the study was given by a parent, guardian or participant (if of suitable age and understanding) and assent was granted by younger participants. Each participant received study equipment (table 1, figure 2) to record and synchronise data and was issued a unique anonymous Microsoft log-in account with a strong password (managed by the study team) for logging into the tablet, Fitbit app (Fitbit Inc, San Francisco, USA) and bespoke Fizzyo app. A researcher demonstrated how to use the equipment to participants. These instructions and technical support contact details were provided as a printout and webpage link via QR code.

For peer review only - http://bmjopen.bmj.com/site/about/guidelines.xhtml

Table 1 Study equipment for participants

	ACT sensor	Activity tracker	Tablet computer
**Description**	Bespoke Fizzyo Sensor. Wireless battery powered pressure sensor that attaches to ACT devices to measure pressure during ACT.	Fitbit Alta HR™. Wrist-worn activity tracker with heart rate sensor to record daily physical activity.	Linx™ 12x64 Windows 10 tablet computer. Required to host remote monitoring apps and games.
**Features**	• Microelectromechanical system (MEMS) based piezoresistive sensor.<br>• Two Buttons for power on/off and game control input.<br>• 1MB flash based storage for approximately 227 hours of data.<br>• 7 days use from full charge. Full charge: 70 minutes.	• OLED customisable display with clock face.<br>• Photoplethysmographic PurePulse® technology heart rate sensor.<br>• 3-axis accelerometer movement detection.<br>• Memory capacity for 7 days of full data.<br>• 7 days use from full charge. Full charge: 1-2 hours.	• Intel Atom x5 processor.<br>• 4GB RAM.<br>• Bluetooth 4.0.<br>• Wi-Fi 802.11.<br>• 64GB memory storage.<br>• 5-7 hours use from full charge. Full charge: 3 hours.
**Data collected**	• Time-stamped ACT pressure data (10Hz).	• Heart rate (variable sampling frequency; 6-30 times per minute),<br>• Step count (per minute).	• Extraction and transmission of Fizzyo sensor and Fitbit data via sync with apps.<br>• Gaming data.
**App Details**	**Fizzyo app (FizzyoHub)**<br>• Developed using Visual Studio for Windows 10,<br>• Bluetooth syncing of the Fizzyo ACT sensor .<br>• *Standard phase*, syncing only,<br>• *Feedback phase*, daily and monthly breath count graphs enabled,<br>• *Gaming phase,* games (developed in Unity, hosted in Microsoft store) able to be installed and played.	**Fitbit app (for Windows 10)**<br>• Developed by Fitbit Inc.<br>• Bluetooth syncing of the Alta HR.<br>• Patient facing dashboard displaying daily and historical graphs of step and activity patterns.<br>• Feedback on progress against daily and longer term goals.	**Windows store app**<br>• Required to install and update Fitbit app and Fizzyo ACT games.

At recruitment, baseline assessments of lung function (spirometry: FEV_1, FVC, FEF_{25-75}) and exercise capacity (10m modified shuttle walk test, with a Polar H10™ heart rate monitor and Fitbit) were undertaken. A quality of life questionnaire (Revised CF Questionnaire (CFQ-R)) and a physiotherapy questionnaire are completed by participants with a member of the study team digitally via REDCap. Spirometry is always performed prior to exercise capacity assessment or after at least 1 hour of rest following maximal exertion. All tests are carried out in accordance with the appropriate guidelines These measurements were repeated midway (7-9 months) and will be collected again at the end of the study (16-months, estimated completed in December 2020). Assessments are carried out on days when participants have a clinic appointment or routine hospital admission (but not during an exacerbation).

For peer review only - http://bmjopen.bmj.com/site/about/guidelines.xhtml

Data are pseudonymised, stored and analysed in the secure GOSH Digital Research Environment (DRE, Aridhia Inc, Edinburgh, UK). The DRE is a cloud-enabled, dedicated GOSH secondary use datastore of medical data from electronic patient records, remote monitoring sensors or external databases. Embedded analytic tools will enable approved researchers to perform statistical analysis within the DRE. Data encryption and transfer protocols and data sharing and processing agreements ensure data are processed in a responsible, lawful, secure and confidential way compliant with NHS information governance standards, the UK data protection act (2018) and the EU GDPR (2018).

Remote monitoring

Activity tracker

A Fitbit activity tracker was chosen as Fitbit Inc. allow developers to use an application programming interface (API) for extraction of processed granular heart rate and step count data. The specific activity tracker model, the Fitbit Alta HR (released in 2017), was chosen with input from CYPwCF and their families at GOSH, from a range of Fitbit devices. It is an appropriate size for the wrists of children from 6 years old and is simple to use.

The Fitbit has a photoplethysmographic heart rate sensor for heart rate measurement, which is essential to physiologically quantify adherence to recommended minutes in daily moderate to vigorous physical activity (MVPA). Heart rate data are collected continuously with a variable sampling frequency (table 1) whenever the Fitbit is worn during the 16 months. Participants are asked to wear the Fitbit during all waking hours except when bathing/swimming (it is not waterproof), and to sync data daily via Bluetooth using the Fitbit app, or at a minimum once per week: the memory capacity of the tracker. Participants are asked to charge the battery at least once per week or when notified by the device/app.

The standard Fitbit app provides feedback data, which is available for participants to view throughout the study. After participants sync their device, the study team extract anonymised data from the Fitbit cloud using the API. It is downloaded to the Fizzyo data cloud and then transferred securely into the DRE (figure 2).

ACT sensor

A bespoke wireless sensor was developed for Project Fizzyo with involvement from CYPwCF and their families at GOSH, physiotherapy specialists and product design specialists. The sensor is battery powered and clips on to compatible ACT devices (figure 3) in the same way that pressure manometers are clipped in during ACT training or use. It measures air pressure changes inside the physiotherapy ACT device during breathing.

Participants are instructed to use the sensor to record all ACT treatments during the study. To reduce the chance of data loss, participants are asked to sync data once per day using the Fizzyo app and charge the sensor at least once per week. Pressure data are stored on the sensor's encrypted memory chip (capacity 1Mb) and can be synchronised either immediately after treatment or stored to sync at a later time. Immediately upon syncing data to the Fizzyo cloud (figure 2) basic breath count processing occurs, to provide breath count feedback to participants within a few seconds (during the feedback phase of the study only). Detailed breath count analysis will be performed on cleaned pseudonymised data in the DRE.

ACT-driven gaming

For peer review only - http://bmjopen.bmj.com/site/about/guidelines.xhtml

Windows compatible games have been developed using a bespoke structured gaming framework (https://github.com/Fizzyo/FizzyoFramework-Unity) in Unity (Unity Technologies, San Francisco, USA), through collaboration with Microsoft, UCL and Abertay University students and CYPwCF at GOSH. The framework takes real time inputs during ACT treatments (expiratory pressure during breathing and a button press) from the Fizzyo sensor, and uses these to simulate standard joystick inputs.

Five bespoke games with different mechanics are available to participants (including Qubi, Archipelayo and Egg Toss); including gamification features such as anonymous high score boards and achievements. Games were designed to encourage the player to blow in the prescribed breath length and pressure range (slightly extended expiration, 15-20cmH_2O), typically in multiple cycles of 8-10 breath sets with pauses for cough/huff. The number of breaths and cycles in the game can be altered by the player, reflecting the personalised ACT prescriptions of children in the study. Gaming is optional for participants during ACTs, but whether games are played or not, can be identified, including which games are played during specific sessions.

Participant questionnaires

Physiotherapy prescription including: ACT device(s), number of breaths and sets per day, estimated duration of ACT, exercise prescription and self-reported routine physical activities for individuals are collected by participant questionnaires at recruitment, mid-point and study end. An age specific CF questionnaire (CFQ-R: 6 to 11years, 12 to 13years or 14years+) is also administered at these timepoints for all participants. A parental CFQ-R is completed for children under 14years of age. The CFQ-R is a CF-specific measure of quality of life, with domains including respiratory and gastrointestinal symptoms, treatment burden and daily functioning; it is currently the best validated CF-specific patient reported outcome measure,[12].

Clinical data

GOSH clinical data are extracted from electronic patient records (EPIC Systems Corp, Wisconsin, USA), pseudonymised and uploaded directly to the DRE. Historical data from older patient record systems (that is not available in electronic patient records) will be extracted, pseudonymised and uploaded to the DRE separately. Where possible, this dataset will contain a participant's entire clinical record including physiotherapy and known key features for CF recorded in the UK CF Registry,[13] such as: genotype, anthropometry, medications, hospital admissions, lung function, pancreatic status, microbiology, co-morbidities, ACT prescription (including documented changes to technique) and exercise test results.

At RLH and RBH electronic patient records are not currently available and clinical information, including demographics and ACT prescription will be extracted from clinical records and databases. Data will be pseudonymised at source, and transferred by secure file transfer protocol to the DRE.

A clinical data processing pipeline will be developed to summarise features describing patient demographics and clinical status as frequencies (e.g. number of hospital admissions) or rates (e.g. number of hospital admissions per year).

DATA ANALYSIS

Adherence to physiotherapy

For peer review only - http://bmjopen.bmj.com/site/about/guidelines.xhtml

The primary outcome measures are: adherence to a participant's daily ACT prescription (during different study phases: standard, feedback, gaming), and adherence to daily physical activity recommendations. ACT sensor (breath pressure) and Fitbit (heart rate, step count) data are processed via a data pipeline in R,[14] within the DRE to describe these outcomes. Clinical data outcomes will be used to investigate any relationship between physiotherapy adherence and specific clinical profiles.

Adherence to medical treatment is the extent to which a patient follows the recommendations agreed upon with health professionals,[15]. As physiotherapists give personalised ACT prescriptions of varying breath count, set count and treatment session number per day, personalised adherence scores will be calculated. Daily ACT adherence will be quantified by the number and type of breaths recorded in a treatment session and per day. Cumulative, aggregate and weekly average scores will also be calculated. Adherence to the prescribed breath count (proportion of completed breaths against prescribed breaths per treatment (usually around 100 breaths)), prescribed breath pressures (proportion of ideal breath pressure breaths (15-20cmH_2O) per treatment) and prescribed breath length (proportion of expiratory breaths at ideal length (at least 1-2seconds) and also the presence of gaps for huff and cough between sets will be assessed. A composite adherence score based on these parameters combined per session, per day and per week will be calculated.

WHO guidelines recommend that all "*children and youth aged 5-17years should accumulate at least 60minutes of MVPA daily*,[16]. Adherence will be measured against this target for all participants. Time in MVPA is calculated daily as time where heart rate is greater than a personalised threshold value (usually around 120 beats per minute), which will consider an individual's resting heart rate, and maximal heart rate from the exercise test). A number of published approaches,[17] are being investigated to determine the optimal MVPA heart rate threshold as no clear approach currently exists in the literature for the study population. Step count data will be used to support heart rate data and assist with identification of sedentary time (for resting heart rate calculation) but will not be the primary method for activity estimation as some activities do not generate a high step frequency despite being vigorous activities (e.g. cycling).

Data Processing Pipeline

A data processing pipeline enabling large amounts of remotely monitored sensor data to be processed efficiently, reliably, and reproducibly was developed in collaboration with Microsoft computer and data science engineers. It was developed using preliminary data collected in the first three months of data collection (September to December 2018) and then tested on a larger dataset (to April 2019).

The pipeline ensures data are processed consistently and data validation checks at each stage alert to errors. The pipeline is being refined throughout the ongoing data collection period but, briefly, it processes data through 3 main steps: *data cleaning* to remove errors, *data labelling* to mark and measure predefined constructs from raw data (for example distinct breaths from pressure traces) and finally *data featurisation*, the quantification of variables for cluster analysis. These steps and the main features for analysis are summarised in table 2.

Statistical analysis

The large amount of heterogeneous data to be recorded and collected for each participant will be analysed using R and visualized using R Shiny apps. Any changes in adherence to physiotherapy prescriptions or recommendations over time or in relation to ACT gaming or feedback, will be quantified. As CF phenotypes and physiotherapy adherence are complex and multifactorial, cluster

For peer review only - http://bmjopen.bmj.com/site/about/guidelines.xhtml

analysis will define groups of individuals based on measured characteristics to identify subgroups of participants with distinct physical activity and/or ACT adherence profiles.

The Fitbit and ACT sensor dataset currently contains 50+ variables (key features shown in table 2), with more to be added from ongoing clinical record extraction. A correlation analysis of the features describing physiotherapy behaviours will remove features which are highly correlated. Variables with a Gaussian distribution will be normalised. Dimensionality reduction will be performed via principal component analysis. These methods will identify the most relevant and independent variables for cluster analysis to ensure a robust definition and visualisation of clusters.

Multiple features of ACT treatment sessions or activity days identified from the principal component and correlation analyses will be grouped using unsupervised cluster analysis, such that both the within-cluster similarity and between-cluster dissimilarity are strong. Unsupervised cluster analysis with the k-means method,[18] is a popular clustering technique and involves grouping multifactorial data into a pre-specified number of clusters. Numerous rapid computations comparing the relationship between multiple variables will determine the optimal cluster centres with the best separation between groups. The silhouette metric will be used to assess cluster homogeneity and separation, to identify the optimal number of clusters.

The associations, patterns and trends of individual physiotherapy adherence clusters to health outcomes (e.g. lung function, number of hospital admissions, infections, antibiotics etc) will be investigated with data from clinical records. Individual participant changes between clusters over time, or in relation to gaming or feedback, will also be investigated.

For peer review only

bmjopen-2020-039587.R1

Table 2: Data pipeline stages and features for cluster analysis

**Sensor**<br>Type of data	**Data pipeline step**			
	**Cleaning**	**Labelling (of)**	**Featurisation** of variables for cluster analysis	
			Descriptive	Describing Adherence$^+$
**ACT Sensor**<br>Pressure-Time	• Removal of blank, duplicate and non-physiological values.<br>• Any non-linear baseline drift corrected, using a sparsity based de-noising approach,[19].	• Treatment sessions<br>• Pressure peaks<br>• Breaths<br>• Breaks between breaths<br>• Sets of breaths	• If any breaths recorded on a day Y/N<br>• Breath count*<br>• Breath length*<br>• Breath peak pressure*<br>• Treatment duration*<br>• Number of treatments per day<br>• Number of sets in a treatment *<br>• Number of breaths per set*	• Adherence score (proportion of days with any breaths recorded per total number of days)<br>• Breath count adherence (proportion of completed breaths against prescribed breaths per treatment)<br>• Set adherence (proportion of sets against prescribed sets per treatment)<br>• Treatment session adherence (proportion of completed treatments against prescribed treatments)<br>• Pressure adherence (proportion of ideal expiratory pressure breaths per treatment)<br>• Breath length adherence (proportion of expiratory breaths at ideal length per treatment)
**Fitbit**<br>Heart rate and step count	• Removal of erroneous or non-physiological data (caused by improper wearing, depleted battery, full chip memory capacity due to infrequent syncing).<br>• Heart rate sampling frequency made consistent (per minute) using a rolling average	• Gaps in data<br>• Wear time (from heart rate data)<br>• Awake wear time<br>• Time in MVPA using personalised heart rate cut off value [17]<br>• Points crossing MVPA cut off value	• Heart rate<br>○ Resting[20]<br>○ Peak<br>○ Density and variability<br>○ MVPA threshold switches<br>• Step count<br>○ Daily step count<br>○ Density and variability<br>○ Active minutes >threshold value<br>• Combined<br>○ Active minutes both heart rate and step count.<br>○ Step count during periods of MVPA	• Daily time in MVPA compared to 60 daily minutes recommended.<br>• Weekly time in MVPA, 7-day window compared to 420 weekly minutes recommended.

Not all features shown. $^+$ Features quantified as adherence per prescribed treatment session, per day, per rolling 7 day week, or other time point as required for analysis. ACT prescription taken from clinical records and physiotherapy questionnaires. * Total and/or average per treatment, standard deviation, min, max values

For peer review only - http://bmjopen.bmj.com/site/about/guidelines.xhtml

Ethics and Dissemination

Ethical approval for this study (IRAS ref: 228625) was granted by the London-Brighton and Sussex, NREC (18/LO/1038). This study is also registered with the GOSH/UCL GOSICH joint research and development office (ref: 17IA06) and has been adopted into the NIHR Clinical Research Network. Study findings will be disseminated via peer-reviewed publications in open source journals, relevant national and international conferences and via CF clinical and patient networks. Results and the statistical code will be published in the Fizzyo GitHub repository and the dataset stored and available through the GOSH DRE. Collaboration with Microsoft has produced two high quality videos already available on YouTube and the production of further high quality resources to disseminate results to both the CF population and participants in the study is planned.

Author contributions

EM, MB, HD, HS, GS, LS conceived and designed the study. GS perceived and developed the sensor and with LS and TK the data processing infrastructure including the bespoke app. EM, ER, HD developed the protocol with input from all investigators. ER, HD, NM, RC recruited and support all participants. JB is the DRE data steward and facilitator of electronic patient record extraction. KK, NF, OL, TVS, BF developed the data processing pipeline and preliminary analysis with EM, GD, NM, RC, HD, MB providing clinical expertise for featurisation. ER, KK, EM, NF, HD will conduct the data analyses. ER and EM wrote the first draft of the manuscript, all authors revised and reviewed this and approved the final manuscript.

Acknowledgements

With thanks to all participants and their families, the clinical CF Teams at each of the recruiting sites, the GOSH DRE team and Microsoft UK, especially Haiyan Zhang, Simon Jackson, and the Microsoft CSE including Josh Lane, Pete Roden, Stephanie Marker, Christian Robles, Kristjana Popovski, Hannah Kennedy and Kristin Ottofy. Also Ryan White, Michael Woollard and Alan Bannon for electronic engineering input, Dean Mohamedally and UCL students (computer science), and Jamie Bankhead and the team at Abertay University for gaming designs and UCL physiotherapy MSc students. We thank Trudell Medical for an ACTNow education award (October 2019).

Funding

This work was supported by the UCL Rosetrees Stoneygate prize (M712), a Cystic Fibrosis Trust Clinical Excellence and Innovation Award (CEA010), A UCL Partners award and the HEFCE Higher Education Innovation Fund (KEI2017-01-04). GD is supported by a Wellcome Institutional Strategic Support Fund award at UCL, and formerly an NIHR Clinical Trials Fellowship. HD is funded by the CF Trust Youth Activity Unlimited SRC and NIHR GOSH BRC internship. All work at UCL GOSICH is supported by the NIHR GOSH BRC. The views expressed are those of the authors and not necessarily those of the NHS, the NIHR or the Department of Health. The study is sponsored by UCL. The funders and sponsor played no role in the design of the study.

Conflicts of interest

GD reports personal lecture fees from Chiesi Limited for an invited talk on Project Fizzyo at an educational event. All other authors report no conflicts of interest in relation to this protocol.

For peer review only - http://bmjopen.bmj.com/site/about/guidelines.xhtml

Protocol registration

Registered with ISRCTN (ISRCTN51624752). We used the SPIRIT checklist when writing our report.

References

1. Keating D, Marigowda G, Burr L, et al. VX-445–Tezacaftor–Ivacaftor in Patients with Cystic Fibrosis and One or Two Phe508del Alleles. New England Journal of Medicine. 2018;379(17):1612-20.
2. Smyth AR, Bell SC, Bojcin S, et al. European Cystic Fibrosis Society Standards of Care: Best Practice guidelines. J Cyst Fibros. 2014;13 Suppl 1:S23-42.
3. Van der Schans CP, Prasad A, Main E. Chest physiotherapy compared to no chest physiotherapy for cystic fibrosis. Cochrane Database of Systematic Reviews. 2000(2).
4. Davies G, Rowbotham NJ, Smith S, et al. Characterising burden of treatment in cystic fibrosis to identify priority areas for clinical trials. J Cyst Fibros. 2020;19(3):499-502.
5. James Lind Alliance Priority Setting Partnership. Cystic Fibrosis Top 10 Priorities [Webpage]. 2017 [Available from: http://www.jla.nihr.ac.uk/priority-setting-partnerships/cystic-fibrosis/top-10-priorities.htm Accessed May 2020.
6. Mikesell CL, Kempainen RR, Laguna TA, et al. Objective Measurement of Adherence to Out-Patient Airway Clearance Therapy by High-Frequency Chest Wall Compression in Cystic Fibrosis. 2017;62(7):920-7.
7. Main E. What is the best airway clearance technique in cystic fibrosis? Paediatric Respiratory Reviews. 2013;14:10-2.
8. Wilson LM, Morrison L, Robinson KA. Airway clearance techniques for cystic fibrosis: an overview of Cochrane systematic reviews. Cochrane Database of Systematic Reviews. 2019(1).
9. Schneiderman JE, Wilkes DL, Atenafu EG, et al. Longitudinal relationship between physical activity and lung health in patients with cystic fibrosis. European Respiratory Journal. 2014;43(3):817-23.
10. Bradley JM, Moran FM, Stuart Elborn J. Evidence for physical therapies (airway clearance and physical training) in cystic fibrosis: An overview of five Cochrane systematic reviews. Respir Med. 2006;100(2):191-201.
11. Tech4good awards: Fizzyo- Gaming technology that stopped the nagging [webpage] 2017 [Available from: https://www.tech4goodawards.com/finalist/fizzyo/ accessed July 2020.
12. Ronit A, Gelpi M, Argentiero J, et al. Electronic applications for the CFQ-R scoring. Respiratory Research. 2017;18(1):108.
13. UK Cystic Fibrosis Registry: Annual Data Report 2018 - Cystic Fibrosis Trust. 2019.
14. R Core Team: R: a language and environment for statistical computing computer program, version 3.5. 0. R Core Team Vienna, Austria; 2018.
15. World Health Organization: Adherence to long-term therapies: evidence for action Geneva: World Health Organization; 2003.
16. World Health Organization. 4, Recommended population levels of physical activity for health. In: Global Recommendations on Physical Activity for Health. Geneva: World Health Organization; 2010.
17. Douglas H, Raywood E, Kapoor K, et al. ePS3.06 Quantifying moderate to vigorous physical activity using heart rate in children and young people with cystic fibrosis. Journal of Cystic Fibrosis. 2019;18:S46.
18. Ding C, He X. K-means clustering via principal component analysis. Proceedings of the twenty-first international conference on Machine learning; Banff, Alberta, Canada: Association for Computing Machinery; 2004. p. 29.
19. Ning X, Selesnick IW, Duval L. Chromatogram baseline estimation and denoising using sparsity (BEADS). Chemometrics and Intelligent Laboratory Systems. 2014;139:156-67.

For peer review only - http://bmjopen.bmj.com/site/about/guidelines.xhtml

20. Kapoor K, Raywood E, Douglas H, et al. Determining resting heart rate in children using wearable activity monitors. European Respiratory Journal. 2019;54(suppl 63):PA342.

Figure Legends

Figure 1: Study design flowchart of the 16 month study per participant. Participant assessment visits are in shaded boxes. Airway clearance techniques (ACTs) and physical activity are recorded daily using electronically chipped devices (a bespoke ACT sensor and a Fitbit activity tracker). The specially developed Fizzyo app, as well as syncing ACT data throughout the 16 months, gives participant feedback in months 2-14 and ACT-driven gaming is available in months 4-12.

Figure 2: Project Fizzyo data collection pathway. Remote monitoring sensors (Fizzyo sensor, Fitbit) connect via Bluetooth to sync data with device specific apps on a study tablet. Anonymous data is sent to the Fizzyo cloud (either directly or via application programming interface from the Fitbit cloud) and then linked with de-identified clinical records in the Great Ormond Street Hospital Digital Research Environment.

Figure 3: Fizzyo sensor and connectors attached to four airway clearance devices. Left are oscillatory positive expiratory pressure (PEP) devices: an Acapella Choice® (labelled) and Aerobika®. Right shows non-oscillatory PEP devices the Pari PEP™ (top) and, AstraTech PEP® (with a mask). Breath pressure changes within the airway clearance device are recorded by the sensor.

For peer review only

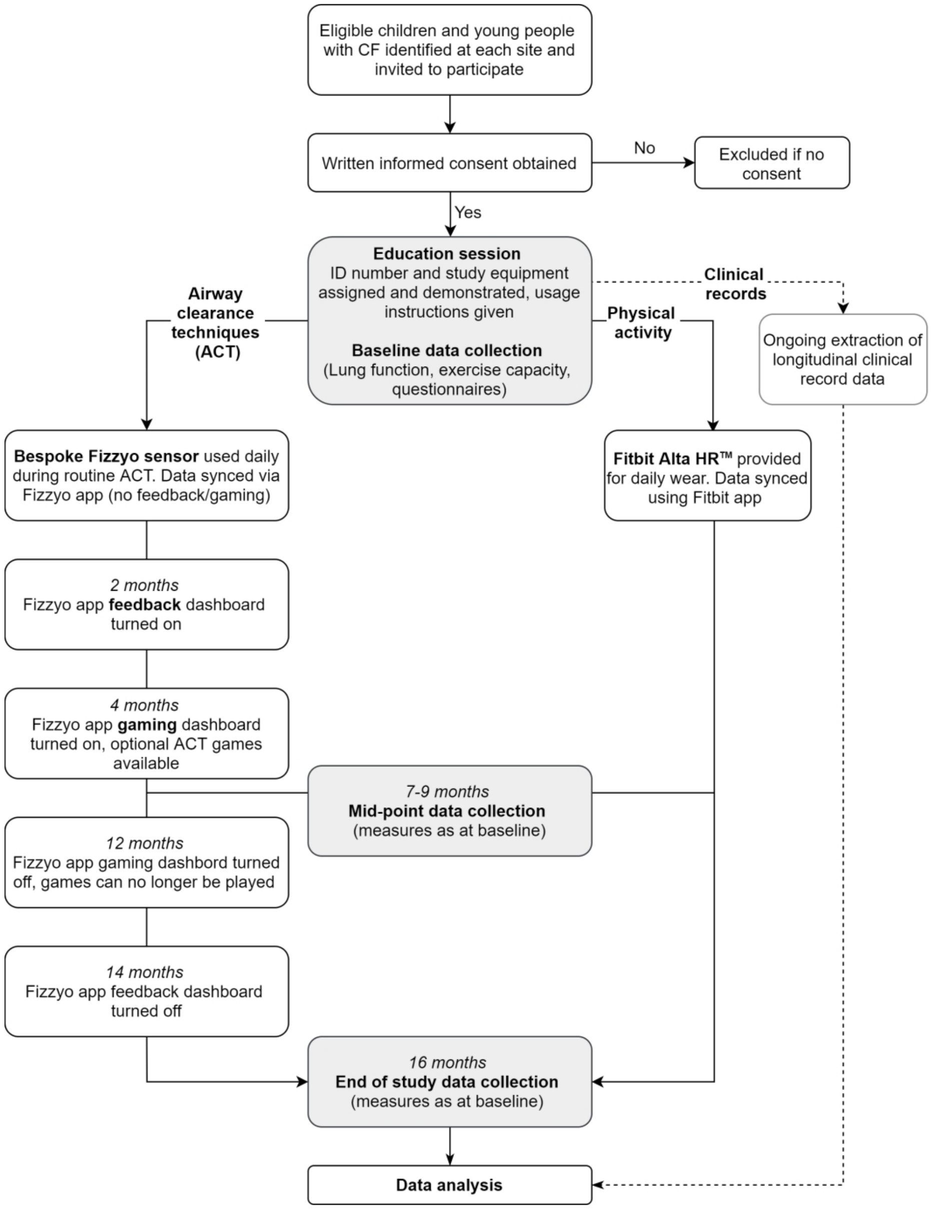

Figure 1: Study design flowchart of the 16 month study per participant. Participant assessment visits are in shaded boxes. Airway clearance techniques (ACTs) and physical activity are recorded daily using electronically chipped devices (a bespoke ACT sensor and a Fitbit activity tracker). The specially developed Fizzyo app, as well as syncing ACT data throughout the 16 months, gives participant feedback in months 2-14 and ACT-driven gaming is available in months 4-12.

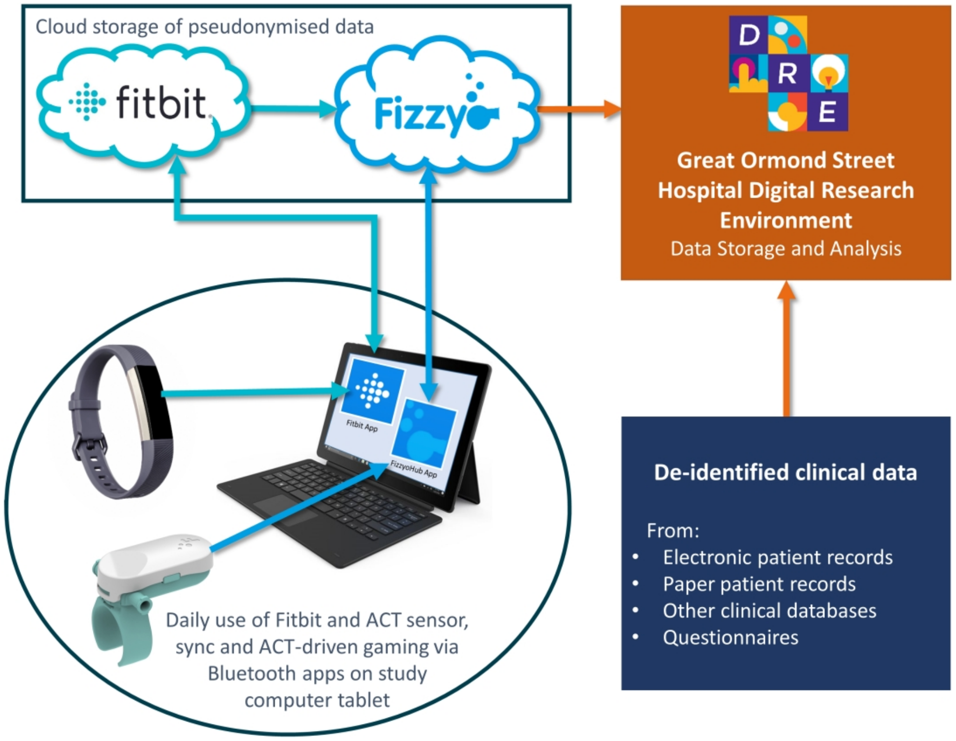

Figure 2: Project Fizzyo data collection pathway. Remote monitoring sensors (Fizzyo sensor, Fitbit) connect via Bluetooth to sync data with device specific apps on a study tablet. Anonymous data is sent to the Fizzyo cloud (either directly or via application programming interface from the Fitbit cloud) and then linked with de-identified clinical records in the Great Ormond Street Hospital Digital Research Environment.

254x190mm (300 x 300 DPI)

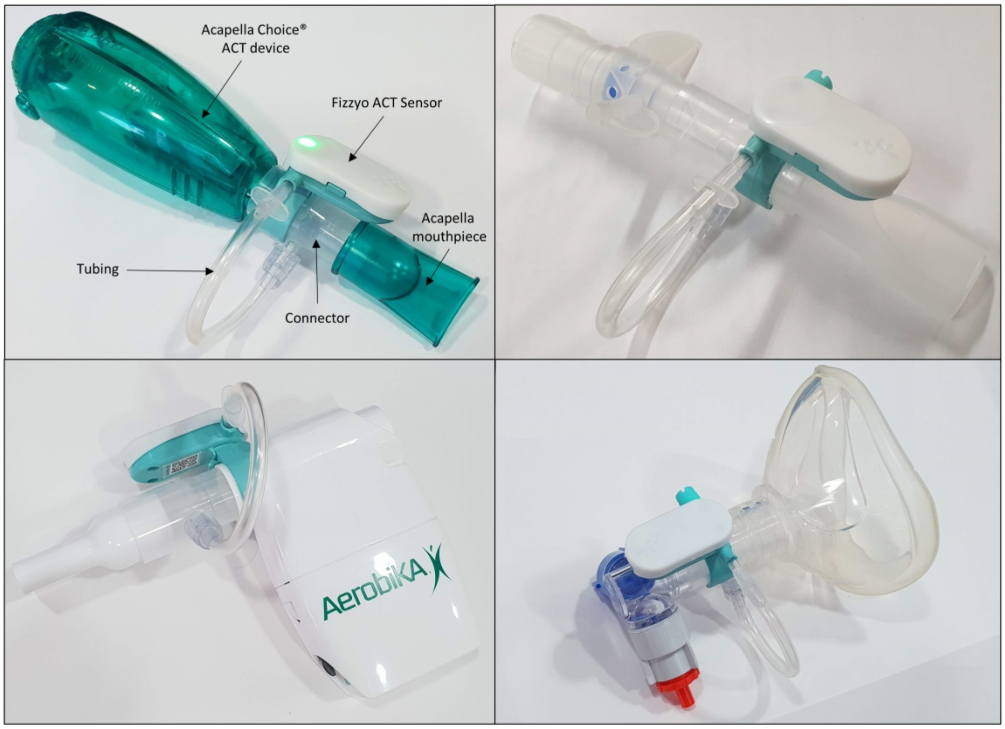

Figure 3: Fizzyo sensor and connectors attached to four airway clearance devices. Left are oscillatory positive expiratory pressure (PEP) devices: an Acapella Choice® (labelled) and Aerobika®. Right shows non-oscillatory PEP devices the Pari PEP™ (top) and, AstraTech PEP® (with a mask). Breath pressure changes within the airway clearance device are recorded by the sensor.

254x190mm (300 x 300 DPI)

Reporting checklist for protocol of a clinical trial.

Based on the SPIRIT guidelines.

Instructions to authors

Complete this checklist by entering the page numbers from your manuscript where readers will find each of the items listed below.

Your article may not currently address all the items on the checklist. Please modify your text to include the missing information. If you are certain that an item does not apply, please write "n/a" and provide a short explanation.

Upload your completed checklist as an extra file when you submit to a journal.

In your methods section, say that you used the SPIRITreporting guidelines, and cite them as:

Chan A-W, Tetzlaff JM, Altman DG, Laupacis A, Gøtzsche PC, Krleža-Jerić K, Hróbjartsson A, Mann H, Dickersin K, Berlin J, Doré C, Parulekar W, Summerskill W, Groves T, Schulz K, Sox H, Rockhold FW, Rennie D, Moher D. SPIRIT 2013 Statement: Defining standard protocol items for clinical trials. Ann Intern Med. 2013;158(3):200-207

		Reporting Item	Page Number
**Administrative information**			
Title	#1	Descriptive title identifying the study design, population, interventions, and, if applicable, trial acronym	1
Trial registration	#2a	Trial identifier and registry name. If not yet registered, name of intended registry	1
Trial registration: data set	#2b	All items from the World Health Organization Trial Registration Data Set	n/a
Protocol version	#3	Date and version identifier	2
Funding	#4	Sources and types of financial, material, and other support	11
Roles and responsibilities: contributorship	#5a	Names, affiliations, and roles of protocol contributors	1, 11

For peer review only

Roles and responsibilities: sponsor contact information	#5b	Name and contact information for the trial sponsor	n/a
Roles and responsibilities: sponsor and funder	#5c	Role of study sponsor and funders, if any, in study design; collection, management, analysis, and interpretation of data; writing of the report; and the decision to submit the report for publication, including whether they will have ultimate authority over any of these activities	11
Roles and responsibilities: committees	#5d	Composition, roles, and responsibilities of the coordinating centre, steering committee, endpoint adjudication committee, data management team, and other individuals or groups overseeing the trial, if applicable (see Item 21a for data monitoring committee)	n/a
**Introduction**			
Background and rationale	#6a	Description of research question and justification for undertaking the trial, including summary of relevant studies (published and unpublished) examining benefits and harms for each intervention	3
Background and rationale: choice of comparators	#6b	Explanation for choice of comparators	8
Objectives	#7	Specific objectives or hypotheses	n/a
Trial design	#8	Description of trial design including type of trial (eg, parallel group, crossover, factorial, single group), allocation ratio, and framework (eg, superiority, equivalence, non-inferiority, exploratory)	4
**Methods: Participants, interventions, and outcomes**			
Study setting	#9	Description of study settings (eg, community clinic, academic hospital) and list of countries where data will be collected. Reference to where list of study sites can be	4

For peer review only

		obtained	
Eligibility criteria	#10	Inclusion and exclusion criteria for participants. If applicable, eligibility criteria for study centres and individuals who will perform the interventions (eg, surgeons, psychotherapists)	4
Interventions: description	#11a	Interventions for each group with sufficient detail to allow replication, including how and when they will be administered	4
Interventions: modifications	#11b	Criteria for discontinuing or modifying allocated interventions for a given trial participant (eg, drug dose change in response to harms, participant request, or improving / worsening disease)	n/a
Interventions: adherance	#11c	Strategies to improve adherence to intervention protocols, and any procedures for monitoring adherence (eg, drug tablet return; laboratory tests)	4
Interventions: concomitant care	#11d	Relevant concomitant care and interventions that are permitted or prohibited during the trial	n/a
Outcomes	#12	Primary, secondary, and other outcomes, including the specific measurement variable (eg, systolic blood pressure), analysis metric (eg, change from baseline, final value, time to event), method of aggregation (eg, median, proportion), and time point for each outcome. Explanation of the clinical relevance of chosen efficacy and harm outcomes is strongly recommended	6-8, table 2
Participant timeline	#13	Time schedule of enrolment, interventions (including any run-ins and washouts), assessments, and visits for participants. A schematic diagram is highly recommended (see Figure)	figure 1
Sample size	#14	Estimated number of participants needed to achieve study objectives and how it was determined, including clinical and statistical assumptions supporting any sample size calculations	4
Recruitment	#15	Strategies for achieving adequate participant enrolment to reach target sample size	4

For peer review only

**Methods: Assignment of interventions (for controlled trials)**			
Allocation: sequence generation	#16a	Method of generating the allocation sequence (eg, computer-generated random numbers), and list of any factors for stratification. To reduce predictability of a random sequence, details of any planned restriction (eg, blocking) should be provided in a separate document that is unavailable to those who enrol participants or assign interventions	n/a
Allocation concealment mechanism	#16b	Mechanism of implementing the allocation sequence (eg, central telephone; sequentially numbered, opaque, sealed envelopes), describing any steps to conceal the sequence until interventions are assigned	n/a
Allocation: implementation	#16c	Who will generate the allocation sequence, who will enrol participants, and who will assign participants to interventions	n/a
Blinding (masking)	#17a	Who will be blinded after assignment to interventions (eg, trial participants, care providers, outcome assessors, data analysts), and how	n/a
Blinding (masking): emergency unblinding	#17b	If blinded, circumstances under which unblinding is permissible, and procedure for revealing a participant's allocated intervention during the trial	n/a
**Methods: Data collection, management, and analysis**			
Data collection plan	#18a	Plans for assessment and collection of outcome, baseline, and other trial data, including any related processes to promote data quality (eg, duplicate measurements, training of assessors) and a description of study instruments (eg, questionnaires, laboratory tests) along with their reliability and validity, if known. Reference to where data collection forms can be found, if not in the	5-7

For peer review only

		protocol	
Data collection plan: retention	#18b	Plans to promote participant retention and complete follow-up, including list of any outcome data to be collected for participants who discontinue or deviate from intervention protocols	n/a
Data management	#19	Plans for data entry, coding, security, and storage, including any related processes to promote data quality (eg, double data entry; range checks for data values). Reference to where details of data management procedures can be found, if not in the protocol	6-7
Statistics: outcomes	#20a	Statistical methods for analysing primary and secondary outcomes. Reference to where other details of the statistical analysis plan can be found, if not in the protocol	8-9
Statistics: additional analyses	#20b	Methods for any additional analyses (eg, subgroup and adjusted analyses)	8-9
Statistics: analysis population and missing data	#20c	Definition of analysis population relating to protocol non-adherence (eg, as randomised analysis), and any statistical methods to handle missing data (eg, multiple imputation)	n/a
**Methods: Monitoring**			
Data monitoring: formal committee	#21a	Composition of data monitoring committee (DMC); summary of its role and reporting structure; statement of whether it is independent from the sponsor and competing interests; and reference to where further details about its charter can be found, if not in the protocol. Alternatively, an explanation of why a DMC is not needed	n/a
Data monitoring: interim analysis	#21b	Description of any interim analyses and stopping guidelines, including who will have access to these interim results and make the final decision to terminate the trial	n/a
Harms	#22	Plans for collecting, assessing, reporting, and managing solicited and spontaneously reported adverse events and other unintended effects of trial interventions or trial conduct	n/a
Auditing	#23	Frequency and procedures for auditing trial conduct, if	n/a

For peer review only

		any, and whether the process will be independent from investigators and the sponsor	
**Ethics and dissemination**			
Research ethics approval	#24	Plans for seeking research ethics committee / institutional review board (REC / IRB) approval	2
Protocol amendments	#25	Plans for communicating important protocol modifications (eg, changes to eligibility criteria, outcomes, analyses) to relevant parties (eg, investigators, REC / IRBs, trial participants, trial registries, journals, regulators)	n/a
Consent or assent	#26a	Who will obtain informed consent or assent from potential trial participants or authorised surrogates, and how (see Item 32)	5
Consent or assent: ancillary studies	#26b	Additional consent provisions for collection and use of participant data and biological specimens in ancillary studies, if applicable	n/a
Confidentiality	#27	How personal information about potential and enrolled participants will be collected, shared, and maintained in order to protect confidentiality before, during, and after the trial	6
Declaration of interests	#28	Financial and other competing interests for principal investigators for the overall trial and each study site	11
Data access	#29	Statement of who will have access to the final trial dataset, and disclosure of contractual agreements that limit such access for investigators	11
Ancillary and post trial care	#30	Provisions, if any, for ancillary and post-trial care, and for compensation to those who suffer harm from trial participation	4
Dissemination policy: trial results	#31a	Plans for investigators and sponsor to communicate trial results to participants, healthcare professionals, the public, and other relevant groups (eg, via publication, reporting in results databases, or other data sharing arrangements), including any publication restrictions	11
Dissemination policy:	#31b	Authorship eligibility guidelines and any intended use of	n/a

For peer review only

authorship		professional writers	
Dissemination policy: reproducible research	#31c	Plans, if any, for granting public access to the full protocol, participant-level dataset, and statistical code	11
**Appendices**			
Informed consent materials	#32	Model consent form and other related documentation given to participants and authorised surrogates	n/a
Biological specimens	#33	Plans for collection, laboratory evaluation, and storage of biological specimens for genetic or molecular analysis in the current trial and for future use in ancillary studies, if applicable	n/a

Notes:

- 12: 6-8, table 2 The SPIRIT checklist is distributed under the terms of the Creative Commons Attribution License CC-BY-ND 3.0. This checklist was completed on 15. April 2020 using https://www.goodreports.org/, a tool made by the EQUATOR Network in collaboration with Penelope.ai

For peer review only
